# The Impact of Paralogy on Phylogenomic Studies – A Case Study on Annelid Relationships

**DOI:** 10.1371/journal.pone.0062892

**Published:** 2013-05-07

**Authors:** Torsten H. Struck

**Affiliations:** Zoological Research Museum Alexander Koenig, Bonn, Germany; Auburn University, United States of America

## Abstract

Phylogenomic studies based on hundreds of genes derived from expressed sequence tags libraries are increasingly used to reveal the phylogeny of taxa. A prerequisite for these studies is the assignment of genes into clusters of orthologous sequences. Sophisticated methods of orthology prediction are used in such analyses, but it is rarely assessed whether paralogous sequences have been erroneously grouped together as orthologous sequences after the prediction, and whether this had an impact on the phylogenetic reconstruction using a super-matrix approach. Herein, I tested the impact of paralogous sequences on the reconstruction of annelid relationships based on phylogenomic datasets. Using single-partition analyses, screening for bootstrap support, blast searches and pruning of sequences in the supermatrix, wrongly assigned paralogous sequences were found in eight partitions and the placement of five taxa (the annelids *Owenia*, *Scoloplos*, *Sthenelais* and *Eurythoe* and the nemertean *Cerebratulus*) including the robust bootstrap support could be attributed to the presence of paralogous sequences in two partitions. Excluding these sequences resulted in a different, weaker supported placement for these taxa. Moreover, the analyses revealed that paralogous sequences impacted the reconstruction when only a single taxon represented a previously supported higher taxon such as a polychaete family. One possibility of *a priori* detection of wrongly assigned paralogous sequences could combine 1) a screening of single-partition analyses based on criteria such as nodal support or internal branch length with 2) blast searches of suspicious cases as presented herein. Also possible are *a posteriori* approaches in which support for specific clades is investigated by comparing alternative hypotheses based on differences in per-site likelihoods. Increasing the sizes of EST libraries will also decrease the likelihood of wrongly assigned paralogous sequences, and in the case of orthology prediction methods like HaMStR it is likewise decreased by using more than one reference taxon.

## Introduction

Molecular phylogenetics has gone through tremendous changes in the last decade with respect to the amount of data used for phylogenetic reconstructions. The shift occurred from using only a single or few specifically chosen genes (e.g., [1], [2], [3], [4], [5], [6], [7], [8], [9]) towards the mining of genomic and transcriptomic data using hundreds of genes (e.g., [10], [11], [12], [13], [14], [15], [16], [17], [18], [19], [20], [21], [22], [23], [24], [25], [26], [27], [28]). The latter approach is also called phylogenomics. The most common approach in phylogenomics is to utilize expressed sequence tags (EST) libraries (e.g., [23], [25], [26], [27], [28], [29]). This means that the transcriptome of a specimen (or tissues of the specimen) is randomly sequenced. The degree of coverage of the transcriptome, thereby, depends among other things on the number of sequence reads; the more reads the better the coverage, but complete coverage is not very likely to be achieved. Hence, which genes are used for the phylogenetic reconstruction is now determined *a posteriori* (after the data generation) and usually each gene is not present in all taxa of the dataset [30]. A crucial step in this *a posteriori* selection process is the determination of orthologous genes across the different libraries of the analysis (e.g., [31]). That is, the sequences of the EST libraries of the different taxa are grouped together into clusters of sequences of the same orthologous gene.

Genes might be related to each other due to different kinds of homology. Two sequences that diverged from each other by a speciation event are called orthologous sequences [32]. Thus, all the members of a set of orthologous sequences can be traced back to a last common ancestor from whom all sequences descended only by speciation events. Orthologous genes allow the reconstruction of speciation events and, hence, the species tree. In contrast, if two sequences diverged from each other by a “gene duplication so that both copies have descended side by side during the history of an organism”, the sequences are called paralogous [32]. Furthermore, paralogous sequences are differentiated into two different kinds. In-paralogs are paralogous sequences of a duplication event that occurred after a speciation event (also called taxon-specific duplication) and out-paralogs are paralogous sequences of a duplication event that occurred before a speciation event. This difference is important with respect to the impact on phylogenetic reconstructions. Generally paralogous genes also reflect the duplication event(s), and thus the gene-family tree is reconstructed and not only the species tree (e.g., [33], [34]). If only one species represents a taxon (e.g., genus, family, or order) and, hence, is the terminal branch on the tree, all duplication events within this taxon are taxon-specific and therefore result in in-paralogs. As all other sequences are orthologous sequences of these in-paralogs the species tree reconstruction will not be affected solely by their paralogous nature. However, if out-paralogous sequences are mistakenly grouped together as orthologous sequences the reconstruction of the species tree can be profoundly confounded, as a gene-family tree is mistaken for the species tree (e.g., [34]).

Determination of orthologous sequences in phylogenomic studies is based on either a bottom-up or a top-down process. In the top-down process a set of orthologous genes is defined usually on the basis of curated databases of orthologous genes derived from taxa for which entire genomes have been already sequenced [26], [27], [29], [35]. This set of genes is often called the core ortholog set and the taxa in this set primer taxa. The genes of this set are searched for in each EST library by employing either blast searches or searches based on hidden Markov models, which are derived from the sequences of the primer taxa in the core ortholog set. Positive hits of this search are then compared against the transcriptome(s) of a reference taxon or taxa using a blast search [31]. Only when this comparison returns the same gene as the best hit, which has been used for the search in the EST library, is the sequence of the library assigned to this gene. This is known as the reciprocity criterion. Programs such as HaMStR [31] implement this strategy.

The bottom-up process does not define a set of orthologous genes *a priori*, but builds clusters of gene families from scratch based on all EST libraries of the analysis (e.g., [23], [25]). Therefore, all EST libraries are pooled and promiscuous conserved domains, which occur in different gene families, are masked in the sequences so that the results of the all-against-all blast searches in the next step is not biased by these domains. The results of the all-against-all blast searches are transferred into a similarity matrix, which is the input for the TribeMCL algorithm. TribeMCL uses graph clustering by flow simulation including iterative and bootstrap procedures to group the sequences into clusters of very similar sequences [36]. The iterative procedure stops when no further improvement in clustering is observed. Important in this aspect is the inflation parameter, as depending on this parameter the clusters can be larger or smaller [36]. The next step is to check if each taxon in a cluster is represented by zero, only one or several sequences [23]. If each taxon is only represented by either one or no sequence the cluster is regarded as being a set of orthologous genes. However, if more than one sequence is found for one taxon it is assumed that paralogous sequences are likely to occur in this cluster. The cluster is either entirely discarded or further analyzed. Therefore, a phylogenetic reconstruction of the affected cluster is conducted to assess the position of the multiple sequence of a taxon. If multiple sequences of a taxon form a monophyletic group within the best tree, it is assumed that the multiple sequences represent in-paralogous sequences, different splice variants or alleles of the same gene or errors in assembly and sequencing. In this case one of the sequences is kept and the others are discarded. However, if multiple sequences of a taxon are placed in different parts of the tree of the affected cluster (i.e., are not monophyletic) this indicates the presence of possible out-paralogous sequences in the cluster. In this case either the entire cluster is discarded [23] or it is broken up into maximally inclusive subclusters [25]; that is, each subcluster has no more than one sequence per taxon and, thus, they supposedly represent the different paralogs of a gene family [25]. Support values for the subcluster or clades within the cluster are not considered in this decision process. Other orthology prediction programs using such a bottom-up approach are, e.g., OrthoMCL, ReMark or MultiMSOAR 2.0 [37], [38], [39].

**Figure 1:**
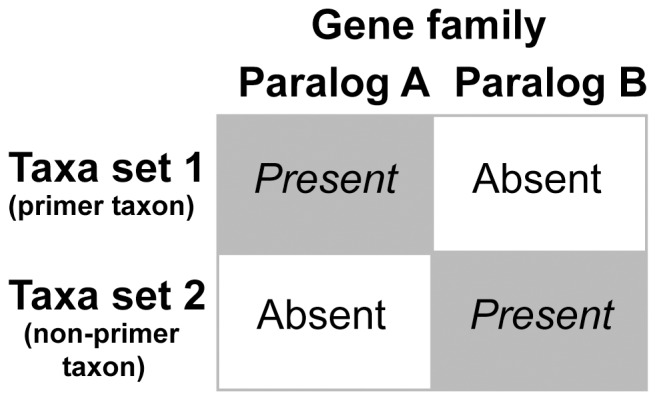
Schematic drawing exemplifying the misleading potential of reciprocal lack in closely related genes. The reciprocal lack of paralogs a and b of gene family A in the two taxon sets can result in a group of supposedly orthologous genes containing both paralogs.

Both procedures are very rigid in their orthology prediction, but in theory both can still erroneously assign paralogous sequences into a single ortholog cluster. This is due to the problem of reciprocal lack (Fig. 1). Consider a gene family with two very closely related paralogs A and B, as similarity among different paralogs of a gene family can be very high. For example, the subunits 1A and 8 of the 70 kDa heat shock protein have e values of 0.0 in reciprocal blast searches and can only be differentiated from each other based on the maximum score (own personal observation). If paralog A is present in one taxon or a set of taxa and paralog B is not, and the opposite applies in another taxon or set of taxa (i.e., B is present and A not), this is called a reciprocal lack. This reciprocal lack can now result in the erroneous assignment of paralogous sequences in both prediction methods. In the top-down approach taxa set 1 of Fig. 1 would be the EST library of a primer taxon or taxa and taxa set 2 the library of the taxon for which the HaMStR analysis is conducted. In course of the prediction a search based on a hidden Markov model for paralog A against the transcriptome of taxa set 2 would recover the sequence of paralog B as the best hit, since paralog A is missing and paralog B is very similar to paralog A. The next step is the blast of this sequence against the EST library of a primer taxon or taxa (taxa set 1 in Fig. 1). Normally, this blast search would return the sequence of paralog B as the best hit and the reciprocity criterion would not be fulfilled, but as the sequence of paralog B is lacking the best hit in this scenario would be the sequence of paralog A as it is very similar to paralog B. Hence, reciprocity is given and the sequence of paralog B kept as being paralog A. Thus, paralogous sequences would be mistakenly grouped together as orthologous sequences.

A similar situation can occur in the bottom-up approach. Consider that for some reason the transcriptomes of one set of taxa (taxa set 1 in Fig. 1) were to contain paralog A, but not the very similar paralog B, and another non-overlapping set of taxa (taxa set 2 in Fig. 1) paralog B, but not A. As these paralogous sequences are very similar to each other, the likelihood is high that the group of these two sets of paralogous sequences are clustered together by the TribeMCL process and not broken apart. If now either no single taxon with multiple sequences is present in this cluster or if multiple sequences are present in a taxon but all of them group together as a monophyletic clade in the best tree of the following phylogenetic reconstruction, given the above mentioned acceptance criteria this cluster would be regarded as set of orthologous sequences. However, indeed it is composed of a set of two paralogous sequences. As can be seen by this explanation the bottom-up approach is less susceptible than the top-down approach as each taxon of the dataset must either fulfill the reciprocal lack criterion or lack both paralogs completely. In contrast, the top-down approach can be one-to-one comparison if only a single reference is chosen for the back-blast procedure of the approach.

Reciprocal lack of paralogs could be due to actual loss of one paralog in one set of taxa and loss of the other one in another set of taxa due to similar selection pressures within each set. However, one would usually expect that several taxa would still possess both paralogs and, thus, distort the pattern. Hence it is more likely that the paralog is not lost and the reason for the reciprocal lack is related to sampling strategies to generate the EST libraries. For example, the sizes of the EST libraries would matter; the smaller the libraries and, thus, the lower the coverage of the transcriptome, the more likely the incorrect annotation of paralogs as orthologs becomes. Other reasons could be that different tissues or organs have been used, which express the paralogs differently. Hence, one paralog is present in one tissue and the other one in the other. This is then also reflected in transcriptomic libraries generated from these different tissues. Such patterns of reciprocal lack can occur if different developmental stages have been used for the construction of the library or the taxa have been exposed to different environmental conditions. For example, some taxa were sampled from habitats within their normal range and others from habitats, which induce strong stress on them. Thus, different aspects can result in the occurrence of reciprocal lacks among different transcriptomic libraries.

Even if such reciprocal lacks occur in a few genes, resulting in erroneously assigned paralogous sequences for different taxa, the question remains: how strong is the impact on the phylogenetic reconstruction in phylogenomics, which is based on more than hundred genes? Will the artificial signal of the gene family tree be cancelled out as noise [40] or will it prevail even in such large-scale analyses [41]? Whereas the impact of artificial signal due to missing data or long branch taxa on phylogenomic studies has been addressed (e.g., [25], [30], [42], [43], [44], [45], [46], [47]), at present it is implicitly assumed that the artificial signal of paralogs is cancelled out, as thorough analyses for paralogy after the orthology determination involving nodal support or branch length assessments are not routinely conducted in phylogenomic studies.

To investigate these questions in a real biological dataset I choose the phylogenomic dataset of Struck et al. [26] addressing the phylogeny of Annelida, plus additionally new data for *Owenia fusiformis* (Annelida). Annelida comprises over 16,500 described species occurring in marine, limnic and terrestrial habitats and is one of the few bilaterian taxa with a segmented body organization. The phylogeny has been controversially discussed for centuries and only little progress has been obtained regarding the relationships beyond the family level using either morphological data (e.g., [48]) or molecular data from a few genes (e.g., [2], [3], [49], [50], [51], [52], [53], [54], [55], [56]). However, a recent phylogenomic study based on 39 taxa and 47,953 amino acid positions derived from 231 genes provides considerable progress towards a robust phylogeny of Annelida [26]. Thus, substantially increasing the number of genes also had a positive impact on the reconstruction of the annelid phylogeny. However, in previous studies the relationships among annelid taxa were so poorly supported at several basal nodes, even using 78 ribosomal genes [56], that the artificial signal of paralogous sequences mistakenly grouped together as orthologs could have had an impact on the analyses even in the case of 230 additional genes. Moreover, more than half of the libraries used by Struck et al. [26] were small, with only about 1,000 high quality reads per taxon. Thus it is more likely that a misleading pattern as described above can be observed. Finally, Struck et al. [26] used a top-down approach for orthology prediction, which is more susceptible to reciprocal lack.

**Figure 2:**
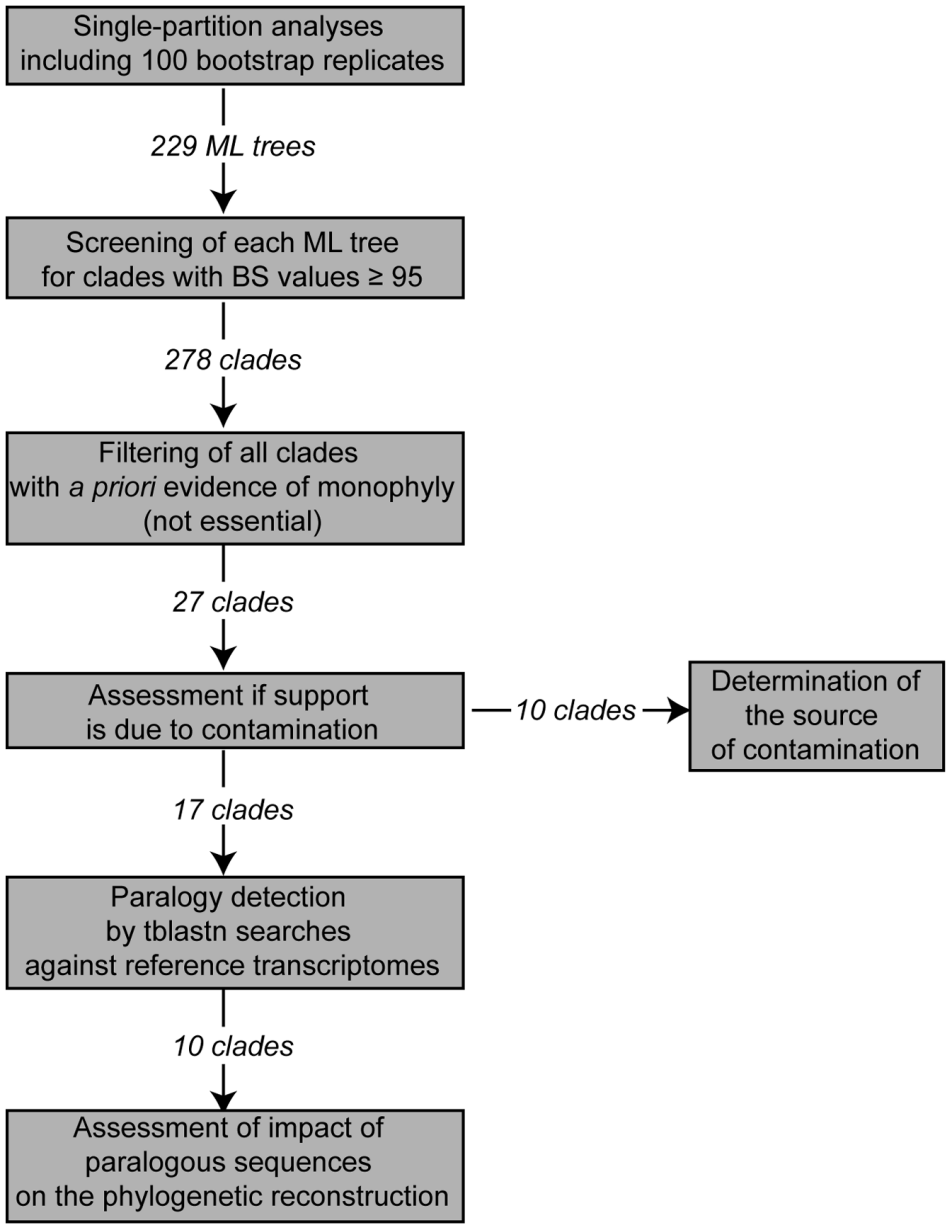
Workflow showing the procedure used herein to detect paralogy. The specific settings for the analyses are also provided.

To detect paralogy after orthology prediction I used a modified procedure from phylogenomic studies addressing eukaryotic or metazoan phylogenies [57], [58], [59]. In these studies single-partition analyses were conducted and all clades above a certain bootstrap value were retrieved and compared against the best tree obtained from the concatenated dataset. Only clades not congruent with the best tree of the concatenated dataset were kept and assessed with respect to paralogy. These assessments comprised considerations based on the tree topology such as nearest neighbor interchanges or long branches as well as blast searches, but it was not clearly stated how these analyses were conducted as well as what priority one type of assessment had over the others. Moreover, to filter against the best tree of the concatenated dataset introduces circularity and strongly reduces the chance to detect clades in the best tree, which are supported mainly by paralogous sequences. On the other hand, although a filtering step is not a prerequisite for this approach, it might be convenient to scale down the number of cases requiring further analysis by manual procedures such as blast searches against NCBI. For example, two species of the genus *Myzostoma* are present in the dataset of Struck et al. [26] as the only representatives of Myzostomidae, and monophyly of Myzostomidae is well established by independent *a priori* evidence from single gene analyses [60]. Thus, it can be safely assumed that a clade of only these two species will be present in many single-partition analyses with strong bootstrap support, which is due to phylogenetic signal and not paralogy. Based on the principle of the above procedure I used the more strict and systematic procedure described here (Fig. 2). First, single-partition analyses were conducted and, second, all clades above a certain bootstrap value detected. Third, all clades congruent with a set of clades, for which strong independent *a priori* evidence of monophyly could be shown, were discarded, but that is not a prerequisite for this approach. As the scope of this manuscript was to find cases which could have had a misleading impact on the analyses of the concatenated data, it seemed reasonable to exclude detected clades with strong *a priori* evidence of monophyly even if this meant to miss some cases of erroneously assigned paralogs. Fourth, the remaining clades were investigated for contamination. Fifth, the sequences of all remaining clades after the fourth step as well as other sequences from the same partition were subjected to blast searches against reference transcriptomes to detect paralogy. All cases of detected paralogy were analyzed with respect to their impact on the phylogenetic reconstruction of the concatenated dataset.

In my analyses concerning the impact of paralogy on phylogenomic studies I found that eight partitions are affected by paralogy in the dataset and that paralogy can have an impact on the phylogenetic reconstruction. In the analyses of the combined data the placement of five taxa (the annelids *Owenia*, *Scoloplos*, *Sthenelais* and *Eurythoe* and the nemertean *Cerebratulus*) and the robust bootstrap support for these placements stemmed from an artificial signal of wrongly assigned paralogous sequences. Excluding these sequences resulted in different placements for these taxa. In contrast, in other cases the exclusion of paralogous sequences had no effect on the placement and support for the position of the affected taxa. Hence, in these cases the phylogenetic signal in the dataset was able to overwhelm the artificial signal of the paralogous sequences. This was always the case when at least one of the taxa was part of a higher taxon (e.g., Clitellata, Terebelliformia, Echiura/Capitellidae, Myzostomidae, Mollusca), which had already gained some support in previous morphological analyses or molecular studies based on a single or few genes. Thus, paralogy can have an impact on phylogenomic studies, but only on taxa whose placement to a sistergroup gains only weak support from the data. Therefore, increasing taxon sampling by including a different species from, for example, the same family or even genus will likely counterbalance this effect as it stabilizes the sistergroup relationship of the affected taxon.

## Materials and Methods

Specimens of *Owenia fusiformis* were collected in the deep channel of the German bight near the North Sea island Helgoland. Collection permits and approvals were not required as *O. fusiformis* is not an endangered marine invertebrate and the collection site was not a protected area. Animals were snap frozen in liquid nitrogen and then stored at −70°C. RNA extraction, EST library preparation and 5′-end sequencing of 1,003 clones using Sanger based sequencing technology were done as described in Struck et al. [26]. Further bioinformatic processing was also conducted as described in Struck et al. [26] including assembly of EST data into contigs, quality trimming of sequences, orthology prediction using either the human ribosomal proteome for local blast searches or the program HaMStR [31], and translation into amino acids using ESTwise [61].

As it is crucial for this study, the orthology prediction is outlined in more detail here. First, the 79 human ribosomal proteins were retrieved from the Ribosomal Protein Gene Database [62] and blast searches against the *O. fusiformis* EST library were conducted. The best hit of each search was kept if the e value was below e^−20^. The settings for the orthology prediction using HaMStR were the same as in Struck et al. [26] to enforce comparability between the studies. However, the new version of HaMStR v8 was used instead of HaMStR v1.4, as there were some critical fixes of bugs in the program. Therefore, the orthology prediction using HaMStR was also repeated for all 39 taxa of the analyses of Struck et al. [26]. The set of core orthologous genes for lophotrochozoans comprised the primer taxa *Helobdella robusta*, *Capitella teleta*, *Lottia gigantea*, *Schistosoma mansoni*, *Daphnia pulex*, *Apis mellifera* and *Caenorhabditis elegans* and all ribosomal proteins in this set were removed to avoid redundancy with the procedure above. For searches using the hidden Markov model against the transcriptomes of *O. fusiformis* as well as of the other 39 taxa the representative option was used instead of the rbh option. The representative option keeps up to three best hits for the blast search against the reference taxon/taxa. If two or more of these hits fulfill the reciprocity criterion and match to non-overlapping parts of the reference protein, the sequences of these hits will be concatenated into a single sequence. Thus, more sequence information is integrated in the final database as opposed to the rbh option, which keeps only the best hit of the search with the hidden Markov model. These blast searches against reference taxa were restricted to *H. robusta*, as the only other annelid of the primer taxa *C. teleta* is known to exhibit strongly increased substitution rates in rRNA genes.

The assembly resulted in 572 contigs/singletons for *O. fusiformis* and 83 out of 231 genes used by Struck et al. [26] were found. The 231 genes were compiled from the individual taxa, aligned using MAFFT [63] and masked using REAP [64] with default settings resulting in 231 aligned and masked partitions. Thus, in this study partition corresponds to a single gene fragment that has been trimmed using REAP. EST data of *O. fusiformis* have been deposited in dbEST (JZ197091–JZ198037) and the all datasets analyzed in this study as well as the scripts used are available at DataDryad (http://dx.doi.org/10.5061/dryad.4js80).

Phylogenetic analyses were conducted on each individual partition using RAxML 7.3.1 [65]. As in Struck et al. [26] for each partition the LG+Γ+I substitution model was chosen as the best fitting model to ensure comparability across all analyses herein and with Struck et al. [26]. Support values of the ML tree were computed using 100 bootstrap (BP) replicates [66]. During a manual inspection to determine whether the script for bootstrap screening (see below) did really find all suspicious cases, I noticed by chance that two partitions had the exact same topology. Therefore, the taxon compositions of all partitions were compared to each other if they had the same composition. For the cases with the same composition the topologies were compared. This revealed another two partitions, which had the exact same topology. Thus, two times two redundant partitions were present in the data of Struck et al. [26]. This was confirmed by a comparison of the sequences in the two partitions. For each taxon the sequences were exactly identical. The first redundancy was between the RPS4x and RPS4y partitions. These are sex chromosome-bound inparalogs of RPS4 within humans [67] and are not present in invertebrates. Therefore, both partitions comprised the very same sequence data. Similarly, the sequence data of partition 22638 was identical to the data of the RPS23 partition. This was due to the fact that Struck et al. [26] used two approaches to compile their dataset. Besides the genes of the lophotrochozoan core ortholog set of the HaMStR approach Struck et al. [26] also used local blast searches with all human ribosomal proteins. However, between these two ortholog sets was an overlap of ribosomal protein genes and they missed this one, 22638, when they deleted the ribosomal genes from the lophotrochozoan core ortholog set. Due to this redundancy the genes RPS4x and 22638 were excluded from the following analyses, which are all based on 229 partitions.

The Maximum Likelihood (ML) tree of each individual partition was screened for groupings of taxa, which might indicate placement not due to phylogenetic signal, but to contamination or paralogy (Fig. 2). Accordingly, the following criterion was invoked. In analyses of single or only a few genes bootstrap support for relationships among annelid taxa beyond the family level is generally low except for a few instances (e.g., [1], [2], [4], [5], [49], [50], [51], [53], [55], [56], [68], [69], [70]). Hence, all clades supported by a significant bootstrap value of 95 or higher were extracted from the trees for further analyses except for the case when the clades comprised only members of either Clitellata, Sipuncula, Myzostomidae, Terebelliformia, Capitellidae/Echiura or Serpulidae (as a representative of Sabellida)/Spionidae. Monophyly of these clades is already well established by independent *a priori* evidence from molecular and/or morphological data (e.g., [2], [48], [51], [60], [71]). Thus, if a significantly supported clade comprised only clitellates it was not considered any further, but if it would additionally comprise the capitellid *Capitella teleta* the clade would be further investigated. Due to this filter only 27 clades (see Tables 1, 2,3, 4) had to be further investigated instead of 278 clades without the filter. Custom perl scripts were written to conduct the screening.

**Table 1:** Results of the contamination and paralogy assessment based on tblastn searches for partitions 21904, 22375, 22431, 22433, and 22539.

partition		species	gene name	e-value	assessment	action taken	comments
21904	A	*U. caupo*	Rho GDP dissociation inhibitor β	e^−62^	potential	partition	Signiature aa present
		*L. gigantea*	Rho GDP dissociation inhibitor β	e^−62^	paralog	excluded	
	B	*M. cirriferum*	Rho GDP dissociation inhibitor α	e^−33^			
		*P. lamarckii*	Rho GDP dissociation inhibitor α	e^−60^			
		*C. gigas*	Rho GDP dissociation inhibitor α	e^−63^			
	PT^1^	*C. teleta*	Rho GDP dissociation inhibitor α	e^−63^			
		*H. robusta*	Rho GDP dissociation inhibitor β	e^−64^			
22375	A	*L. conchilega*			contamination	sequences	identical sequences
		*O. fusiformis*				excluded	
22431	A	*U. caupo*	NSA2 ribosome biogenesis homolog	e^−148^	no paralog	none	
		*C. teleta*	NSA2 ribosome biogenesis homolog	e^−144^			
		*A. marina*	NSA2 ribosome biogenesis homolog	e^−71^			
	PT^1^	*H. robusta*	NSA2 ribosome biogenesis homolog	e^−141^			
		*L. gigantea*	NSA2 ribosome biogenesis homolog	e^−151^			
22433	A	*S. armiger*	Proteasome subunit α2 (PSMA2)	e^−73^	potential	sequences	
		*S. boa*	Proteasome subunit α2 (PSMA2)	e^−98^	paralog	excluded	
		*E. complanata*	Proteasome subunit α2 (PSMA2)	e^−63^			
	PT^1^	*H. robusta*	Proteasome subunit α8 (PSMA8)	e^−135^			
		*L. gigantea*	Proteasome subunit α8 (PSMA8)	e^−128^			
		*C. teleta*	Proteasome subunit α8 (PSMA8)	e^−130^			
22539	A	*L. gigantea*	Succinate dehydrogenase complex subunit D	e^−16^	no paralog	none	
		*C. gigas*	Succinate dehydrogenase complex subunit D	e^−17^			
		*O. fusiformis*	Succinate dehydrogenase complex subunit D	e^−20^			
		*T. transversa*	Succinate dehydrogenase complex subunit D	e^−19^			
	A+B	*Urechis caupo*	Succinate dehydrogenase complex subunit D	e^−13^			
		*C. teleta*	Succinate dehydrogenase complex subunit D	e^−22^			
	PT^1^	*H. robusta*	Succinate dehydrogenase complex subunit D	e^−19^			

1 Primer taxa.

**Table 2:** Results of the contamination and paralogy assessment based on tblastn searches for partitions 22606, 22636, 22680, 22820, and 23018.

partition		species	gene name	e-value	assessment	action taken	comments
22606	A	*O. fusiformis*	Centrin, EF-hand protein 3 (CETN3)	e^−57^	potential	sequences	
		*C. lacteus*	Centrin, EF-hand protein 3 (CETN3)	e^−56^	paralog	excluded	
	PT^1^	*C. teleta*	Centrin, EF-hand protein 2 (CETN2)	e^−104^			
		*H. robusta*	Centrin, EF-hand protein 2 (CETN2)	e^−92^			
		*L. gigantea*	Centrin, EF-hand protein 2 (CETN2)	e^−91^			
22636	A	*M. seymourcollegiorum*	Eukaryotic translation initiation factor 5A2	e^−47^	no paralog	none	
		*A. pompejana*	highly similar to Eukaryotic translationinitiation factor 5A2	e^−57^			
	PT^1^	*C. teleta*	Eukaryotic translation initiation factor 5A2	e^−52^			
		*H. robusta*	Eukaryotic translation initiation factor 5A2	e^−56^			
		*L. gigantea*	Eukaryotic translation initiation factor 5A2	e^−61^			
22680	A	*P. lamarckii*	heat shock 70 kDA protein 1A	e^−166^	potential	sequences	
		*A. pompejana*	heat shock 70 kDA protein 1A	e^−32^	paralog	excluded	
	PT^1^	*C. teleta*	heat shock 70 kDA protein 8	0			
		*H. robusta*	heat shock 70 kDA protein 8	0			
		*L. gigantea*	heat shock 70 kDA protein 8	0			
22820	A	*L. conchilega*			contamination	sequences	identical sequences
		*O. fusiformis*				excluded	
22938	A	*B. neritina*	NADH dehydrogenase (ubiquinone)1α subcomplex subunit 13	e^−18^	no paralog	none	
		*C. gigas*	NADH dehydrogenase (ubiquinone)1α subcomplex subunit 13	e^−26^			
	PT^1^	*C. teleta*	NADH dehydrogenase (ubiquinone)1α subcomplex subunit 13	e^−20^			
		*H. robusta*	NADH dehydrogenase (ubiquinone)1α subcomplex subunit 13	e^−29^			
		*L. gigantea*	NADH dehydrogenase (ubiquinone)1α subcomplex subunit 13	e^−18^			
23018	A	*C. lacteus*	proliferating cell nuclear antigen (PCNA)	e^−92^	no paralog	none	
		*T. transversa*	proliferating cell nuclear antigen (PCNA)	e^−119^			
	PT^1^	*C. teleta*	proliferating cell nuclear antigen (PCNA)	e^−136^			
		*H. robusta*	proliferating cell nuclear antigen (PCNA)	e^−145^			
		*L. gigantea*	proliferating cell nuclear antigen (PCNA)	e^−139^			

1 Primer taxa.

**Table 3:** Results of the contamination and paralogy assessment based on tblastn searches for partitions 23291, 23636, 23680, 23729, 23816, and 24126.

partition		species	gene name	e-value	assessment	action taken	comments
23291	A	*L. conchilega*			contamination	sequences	identical sequences
		*O. fusiformis*				excluded	
23636	A	*H. medicinalis*	NADH dehydrogenase (ubiquinone) 1α subcomplex,assembly factor 2	e^−09^	potential	sequences	
		*A. pompejana*	NADH dehydrogenase (ubiquinone) 1α subcomplex,assembly factor 2	0.043	paralog	excluded	
	PT^1^	*C. teleta*	NADH dehydrogenase (ubiquinone) 1αsubcomplex 12	e^−31^			
		*H. robusta*	NADH dehydrogenase (ubiquinone) 1αsubcomplex 12	e^−34^			
		*L. gigantea*	NADH dehydrogenase (ubiquinone) 1αsubcomplex 12	e^−33^			
23680	A	*G. tridactyla*			contamination	sequences	identical sequences
		*T. pigmentata*				excluded	
23729	A	*P. dumerilii*	trans-2,3-enoyl-CoA reductase (TECR)	e^−104^	no paralog	none	
		*E. clavigera*	trans-2,3-enoyl-CoA reductase (TECR)	e^−96^			
	PT^1^	*C. teleta*	trans-2,3-enoyl-CoA reductase (TECR)	e^−115^			
		*H. robusta*	trans-2,3-enoyl-CoA reductase (TECR)	e^−97^			
		*L. gigantea*	trans-2,3-enoyl-CoA reductase (TECR)	e^−117^			
23816	A	*M. fuliginosus*	Aldolase A	e^−105^	potential	partition	C is only minimally worse
		*C. teleta*	Aldolase C	0	paralog	excluded	A is only minimally worse
	B	*R. piscesae*	Aldolase A	0			C is clearly worse
		*C. gigas*	Aldolase A	0			C is clearly worse
	PT^1^	*H. robusta*	Aldolase C	e^−177^			A is only minimally worse
		*L. gigantea*	Aldolase A	e^−169^			C is only minimally worse
24126	A	*L. gigantea*	xin actin-binding repeat containing 2 (XIRP2)	e^−13^	potential	partition	
		*T. lageniformis*	LIM and SH3 domain protein 1-like	e^−12^	paralog	excluded	
	PT^1^	*H. robusta*	cysteine- and glycine-rich protein 3 (cardiac LIM protein)	e^−15^			
		*C. teleta*	cysteine- and glycine-rich protein 3 (cardiac LIM protein)	e^−09^			

1 Primer taxa.

**Table 4:** Results of the contamination and paralogy assessment based on tblastn searches for partitions RPL13a, RPL15, RPL24, RPS15, RPS24, RPS6 and RPSA.

partition		species	gene name	e-value	assessment	action taken	comments
RPL13a	A	*L. conchilega*			contamination	sequences	identical sequences
		*O. fusiformis*				excluded	
RPL15	A	*L. conchilega*			contamination	sequences	identical sequences
		*O. fusiformis*				excluded	
RPS15	A	*L. conchilega*			contamination	sequences	identical sequences
		*O. fusiformis*				excluded	
RPS24	A	*L. conchilega*			contamination	sequences	identical sequences
		*O. fusiformis*				excluded	
RPS6	A	*L. conchilega*			contamination	sequences	identical sequences
		*O. fusiformis*				excluded	
RPSA	A	*L. conchilega*			contamination	sequences	identical sequences
		*O. fusiformis*				excluded	
RPL24	A	*L. conchilega*	RPL24 domain containing 1	e^−27^	potential	sequences	
		*E. fetida*	RPL24 domain containing 1	e^−28^	paralog	excluded	
	PT^1^	*C. teleta*	RPL24	e^−59^			
		*H. robusta*	RPL24	e^−48^			
		*L. gigantea*	RPL24	e^−50^			

1 Primer taxa.

In a first assessment the 27 clades, which are potentially problematic, were investigated if the strong support could be due to contamination and not paralogy based on two criteria (Fig. 2). First, at least one of the taxa had to have a very short branch length, ideally a zero branch length. Second, the amino acid sequences were identical or nearly identical for the parts the sequences had in common. If both criteria were fulfilled, the sequences of the clade were flagged as contaminated. In a next step I tried to reveal the possible source of contamination. One reason could be that handling errors occurred during the processing of the taxon in the field, in the laboratory or in the bioinformatic pipeline. If that could be excluded, the potential of different biological reasons for cross-contamination such as gut content, symbionts or parasites was assessed given recent knowledge about the biology of the affected taxa.

In a second assessment the remaining clades were tested for paralogy (Fig. 2). Therefore, the affected sequences of the clade as well as those of the primer taxa (PT) in the corresponding partition (i.e., *Lottia gigantea*, *C. teleta* and *Helobdella robusta*) were used for tblastn 2.2.26+ searches in NCBI against the transcriptomes of *Bos taurus*, *Branchiostoma floridae* and *Homo sapiens*. Herein, the transcriptomes of the deuterostomes *B. taurus*, *B. floridae* and *H. sapiens* were used as preliminary unrestricted blast searches against the complete, non-redundant NCBI database returned hits for these taxa across the searches for all tested taxa. This was, unfortunately, not the case for a protostome taxon. A first search was conducted against *B. taurus*. The e values of the best hits were compared across the taxa to reveal whether they returned different gene assignments or not. If that first search did not result in differences in gene assignments, further searches against *B. floridae* and *H. sapiens* were conducted. These additional searches were done to be certain that there is truly no difference in gene assignment even if another transcriptome were used.

The impact of the contaminated and paralog sequences on the phylogenetic reconstruction of the concatenated dataset of 229 genes was assessed using different datasets. First, the 229 genes were concatenated into a supermatrix without pruning any affected sequences or excluding entire partitions (all data = AD). Second, all contaminated sequences were pruned from the AD dataset (contamination pruned = CPr). For example, the sequences of *Lanice conchilega* and *O. fusiformis* were pruned from partition 22375. Third, in turn either all affected sequences of a partition or the entire partition affected by paralogy were pruned from the CPr dataset to assess the individual influence of each paralogy case on the concatenated analysis. If the primer taxa were not affected by paralogy, only the affected sequences were pruned from the partition, but not the entire partition. If, however, a primer taxon was among the sequences affected by paralogy, the entire partition was pruned from the analyses, as this was taken as evidence that this gene of the core ortholog set was already a mixture of paralogous genes and not of orthologous genes. For example, in partition 21904 *L. gigantea* is among the affected sequences and, hence, the entire partition has been excluded (see Tables 1 & 3 and, i.e., 21904, 23816 and 24126). In contrast, in partition 22433 only the sequences of *Scoloplos armiger*, *Sthenelais boa* and *Eurythoe complanata* are affected and, hence, only these three sequences have been pruned from the dataset (see Tables 1, 2,3, 4 and, i.e., 22433, 22606, 22680, 23636 and RPL24). This resulted in eight additional pruned datasets (CPr21904, CPr23816, CPr24126, CPr22433, CPr22606, CPr22680, CPr23636 and CPrRPL24). Fourth, all affected sequences of a partition or the entire partition affected by paralogy (same criteria as before) were pruned from the CPr dataset to assess the combined influence of the detected cases of paralogy on the concatenated analysis (contamination & paralogy pruned = CPPr). Thus, a total of 11 concatenated datasets was generated using FASconCAT [72]. Phylogenetic reconstructions of each dataset were conducted using RAxML 7.3.1 with the LG+Γ+I substitution model, the automatic bootstopping option (-# autoMRE) to a maximum of 1,000 BP replicates [66] and 100 independent best tree searches as did Struck et al. [26]. Finally, the leaf stability index of each taxon was determined for the analyses of the AD, CPr and CPPr datasets using Phyutility [73].

## Results

**Figure 3:**
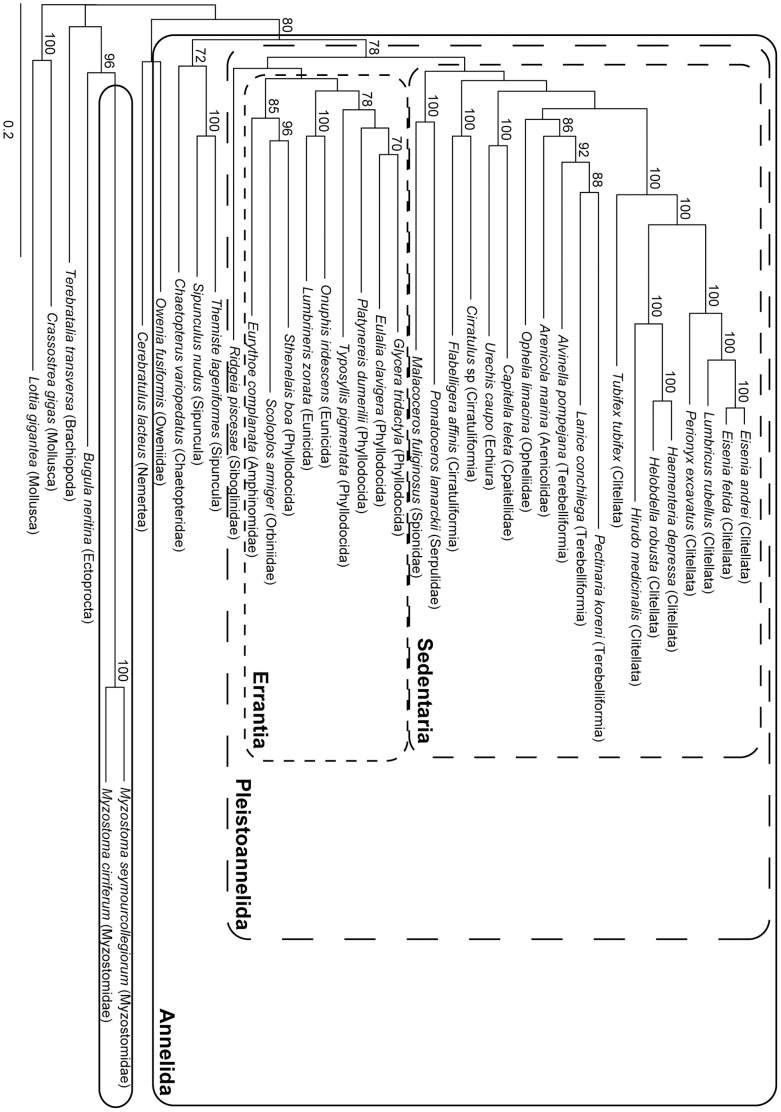
Phylogram of the ML analysis using the AD dataset with no sequences pruned. The dataset comprised 47,848 aa positions (-ln L = 671,889.93). Only bootstrap values ≥70 are shown at the branches. Higher taxonomic groupings of species are provided in brackets behind the species. Scattered boxes indicate Errantia (short lines), Sedentaria (intermediate lines) and Pleistoannelida (long lines) and the solid box Annelida. The scale bar indicates the number of substitutions per site.

The analysis of the concatenated dataset without pruning any sequences (AD dataset) is generally similar to the RAxML analyses of 39 taxa in Struck et al. (Supplementary Fig. 2 in [26]) (Fig. 3). As in their RAxML analyses Myzostomidae showed a long branch attraction to the longest outgroup taxon, *Bugula neritina*, with strong bootstrap support (BP = 96, Fig. 3). Chaetopteridae and Sipuncula were not part of Pleistoannelida and Pleistoannelida was divided into two major clades, Errantia and Sedentaria. In contrast to Struck et al. [26] the siboglinid *Ridgeia piscesae* was sister to Errantia and not placed within Sedentaria, but nodal support was low for this placement herein. In addition, *R. piscesae* was the most unstable taxon in the analyses of Struck et al. [26] and was excluded from their further analyses. Moreover, differences occurred also within Sedentaria with respect to the positions of Opheliidae and Serpulidae/Spionidae, but nodal support for these positions was low in either analysis. Oweniidae, which was not covered by Struck et al. [26], was not part of Annelida, but sister to the nemertean representative, *Cerebratulus lacteus*, with nodal support below 70 (Fig. 3).

**Figure 4:**
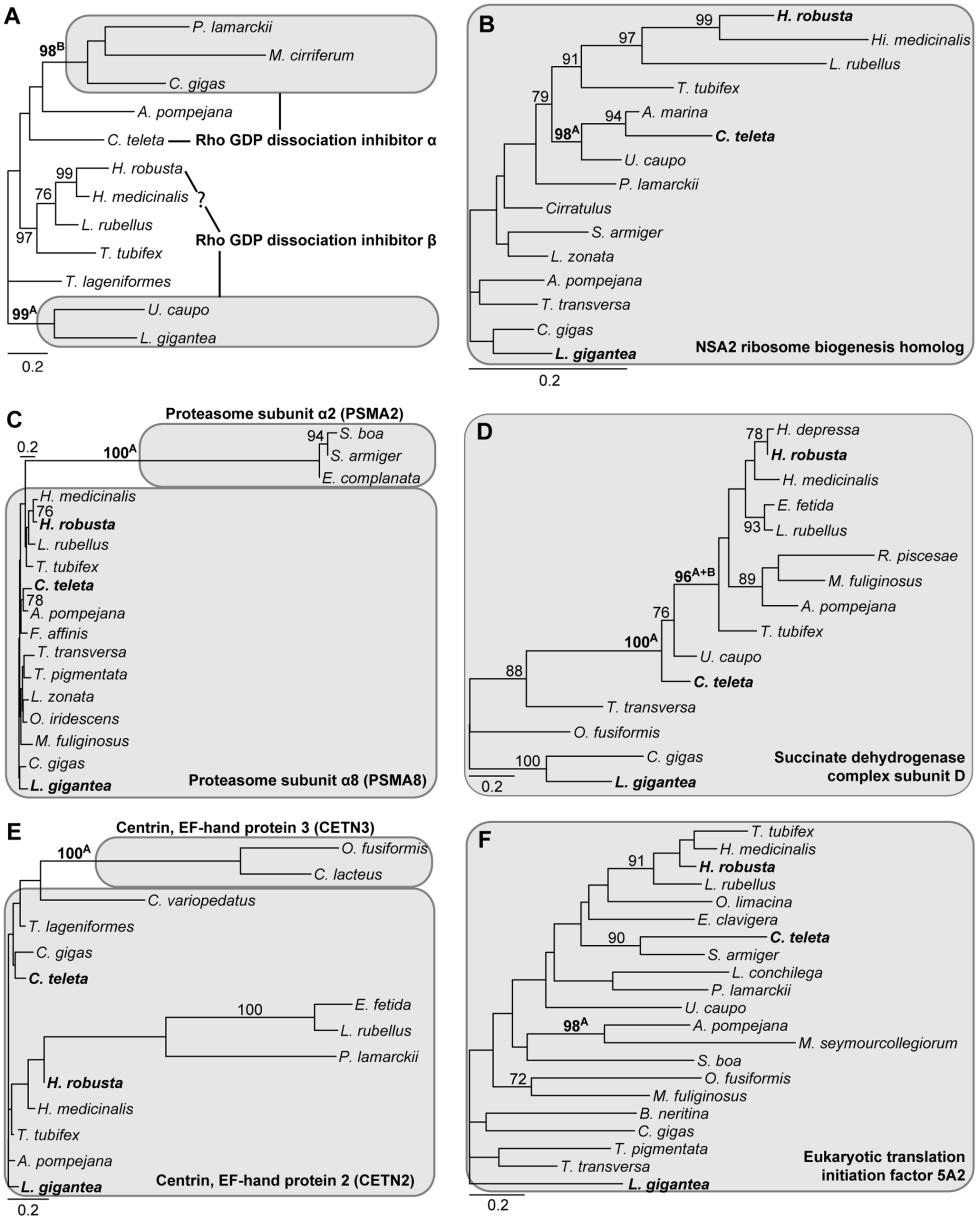
Phylograms of ML analyses of individual partitions. (A) partition 21904, 202 aa positions, 12 taxa, -ln L = 3,077.64. (B) partition 22431, 261 aa positions, 15 taxa, -ln L = 2,410.46. (C) partition 22433, 258 aa positions, 17 taxa, -ln L = 2,794.73. (D) partition 22539, 107 aa positions, 15 taxa, -ln L = 1,751.86. (E) partition 22606, 175 aa positions, 14 taxa, -ln L = 1,998.82. (F) partition 22636, 173 aa positions, 21 taxa, -ln L = 4,121.35. Only bootstrap values ≥70 are shown at the branches. Superscript A and/or B behind bootstrap values refer to the corresponding clades in Tables 1 & 2. Grey boxes indicate the genes with gene names provided. The scale bar indicates the number of substitutions per site. Primer taxa are in bold.

**Figure 5:**
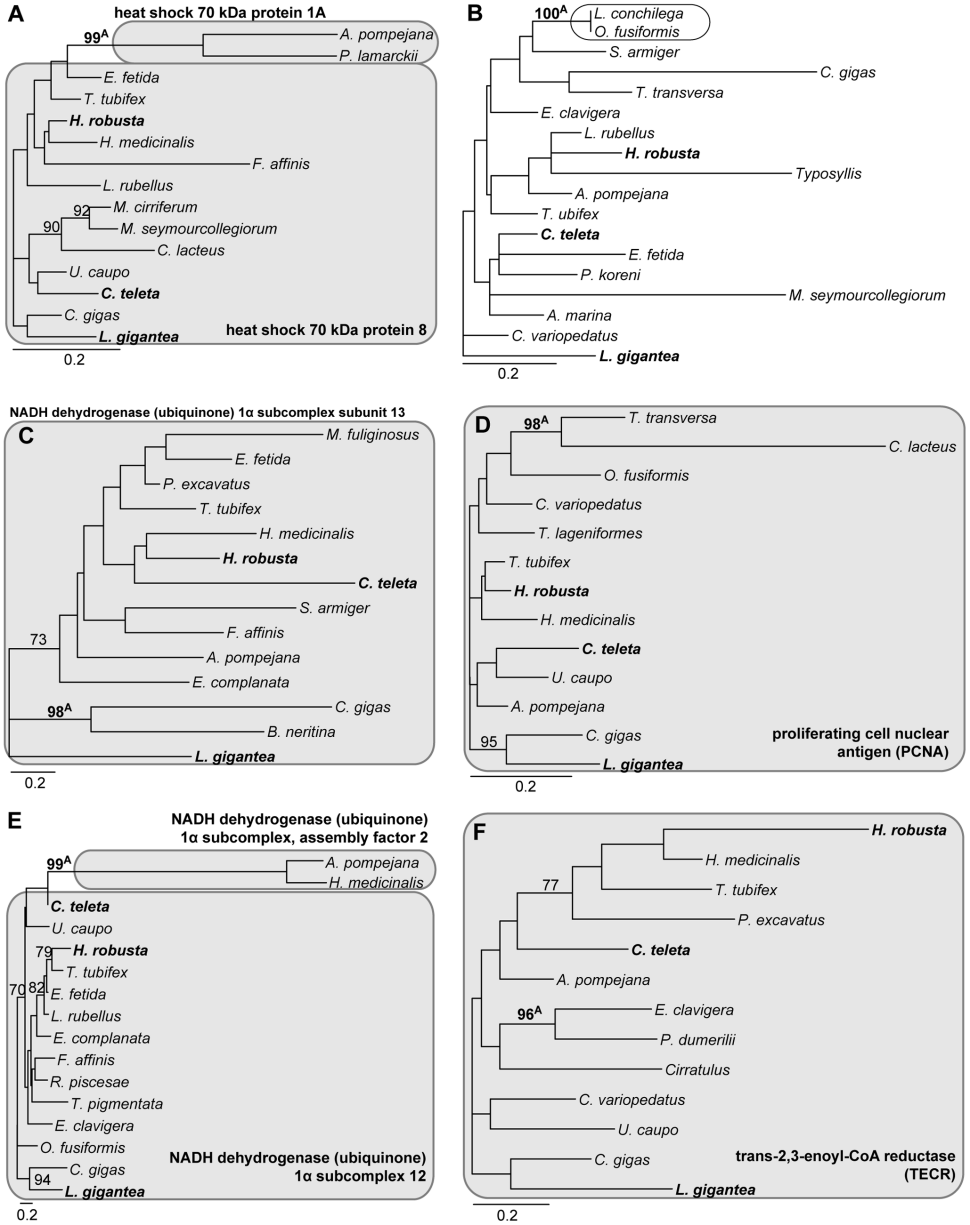
Phylograms of ML analyses of individual partitions. (A) partition 22680, 842 aa positions, 15 taxa, -ln L = 5,717.23. (B) partition 22820, 90 aa positions, 18 taxa, -ln L = 1,308.96. (C) partition 22938, 157 aa positions, 14 taxa, -ln L = 3185.34. (D) partition 23018, 275 aa positions, 13 taxa, -ln L = 2,961.30. (E) partition 23636, 142 aa positions, 16 taxa, -ln L = 2,659.18. (F) partition 23729, 273 aa positions, 13 taxa, -ln L = 3,427.81. Only bootstrap values ≥70 are shown at the branches. Superscript A and/or B behind bootstrap values refers to the corresponding clades in Tables 2 & 3. Grey boxes indicate the genes with gene names provided and the empty one a contamination problem. The scale bar indicates the number of substitutions per site. Primer taxa are in bold.

**Figure 6:**
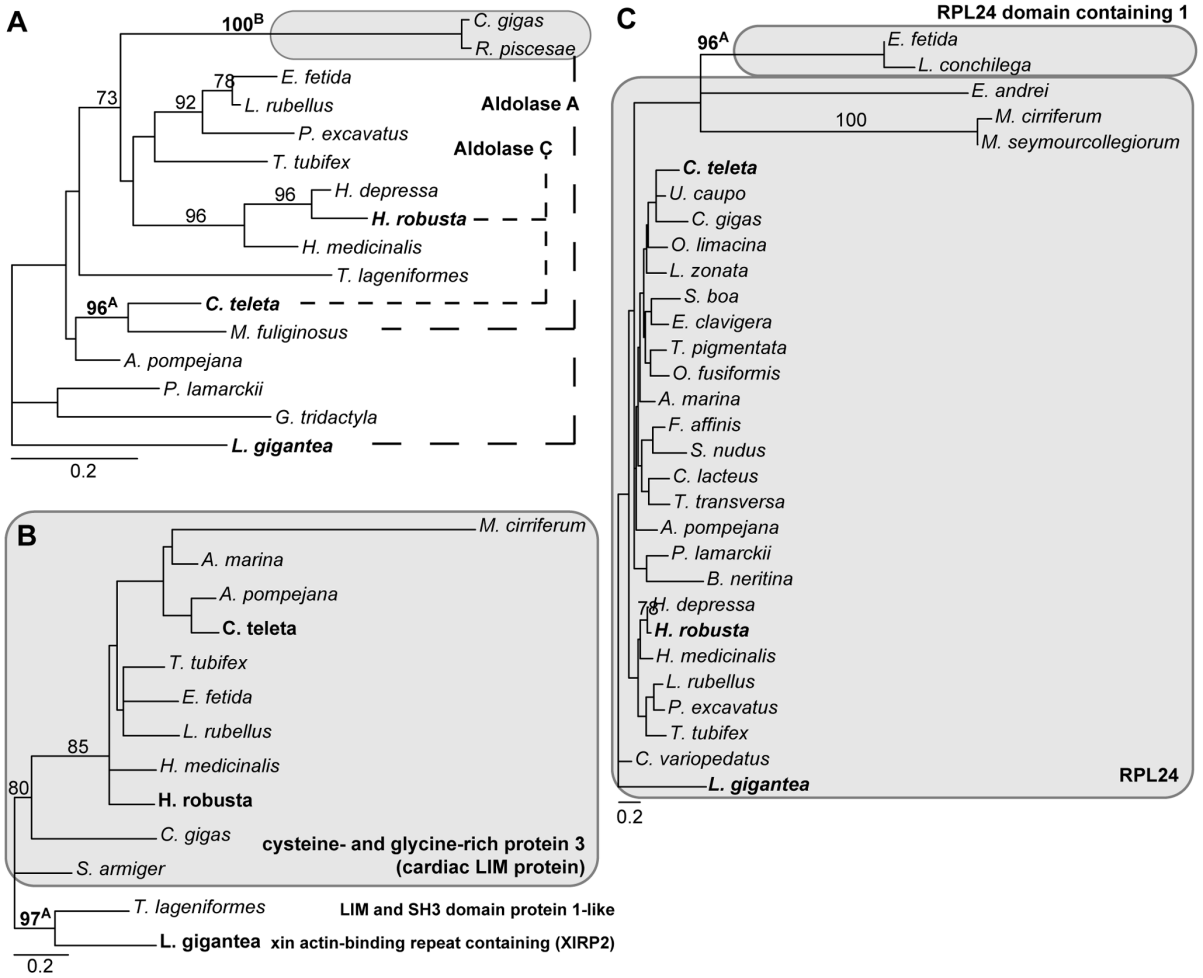
Phylograms of ML analyses of individual partitions. (A) partition 23816, 364 aa positions, 16 taxa, -ln L = 3,707.70. (B) partition 24126, 76 aa positions, 13 taxa, -ln L = 1,058.97. (C) partition RPL24, 162 aa positions, 30 taxa, -ln L = 4,088.56. Only bootstrap values ≥70 are shown at the branches. Superscript A and/or B behind bootstrap values refers to the corresponding clades in Tables 3 & 4. Grey boxes indicate the genes with gene names provided. The scale bar indicates the number of substitutions per site. Primer taxa are in bold.

The screening of the single-partition analyses revealed 27 clades in 24 partitions, which were supported by a bootstrap value of 95 or higher and could not be considered to support monophyly of either Clitellata, Sipuncula, Myzostomidae, Terebelliformia, Capitellidae/Echiura or Serpulidae/Spionidae (Tables 1, 2,3, 4, Fig. 4 Fig. 5 Fig. 6). Closer inspection showed that 10 out of the 27 clades (i.e., partitions 22375, 22820, 23291, 23680, RPL13a, RPL15, RPS15, RPS24, RPS6, and RPSA in Tables 1, 2,3, 4) could be attributed to a contamination problem, as at least one of the taxa showed a zero branch length or very short branch length (e.g., Fig. 5B) and the sequences of the two affected taxa were nearly identical. In all cases the libraries of *Lanice conchilega* and *Owenia fusiformis* were the affected ones except for the one case found in partition 23680, where the libraries of *Glycera tridactyla* and *Typosyllis pigmentata* were affected. A contamination in *Lanice*/*Owenia* due to epibiotic or parasitic life styles or gut content could be excluded. I was able to pinpoint the source of cross-contamination to the handling of the libraries in the sequencing pipeline. In a first run the libraries of the species were processed in parallel in the pipeline for EST sequencing. Moreover, from this run only the library of *L. conchilega* was used, whereas due to other reasons a completely new library was generated for *O. fusiformis*. Hence, in the EST library of *L. conchilega* a small fraction of about 5% is indeed sequence information of *O. fusiformis*. For the one instance of *Glycera* and *Typosyllis* this could be due to gut content, as *Glycera* shows a predatory life style and might have had tissue of a syllid in its gut. These two taxa were never processed in parallel, so that handling errors can be excluded and also both taxa show neither an epibiotic nor a parasitic life style.

**Figure 7:**
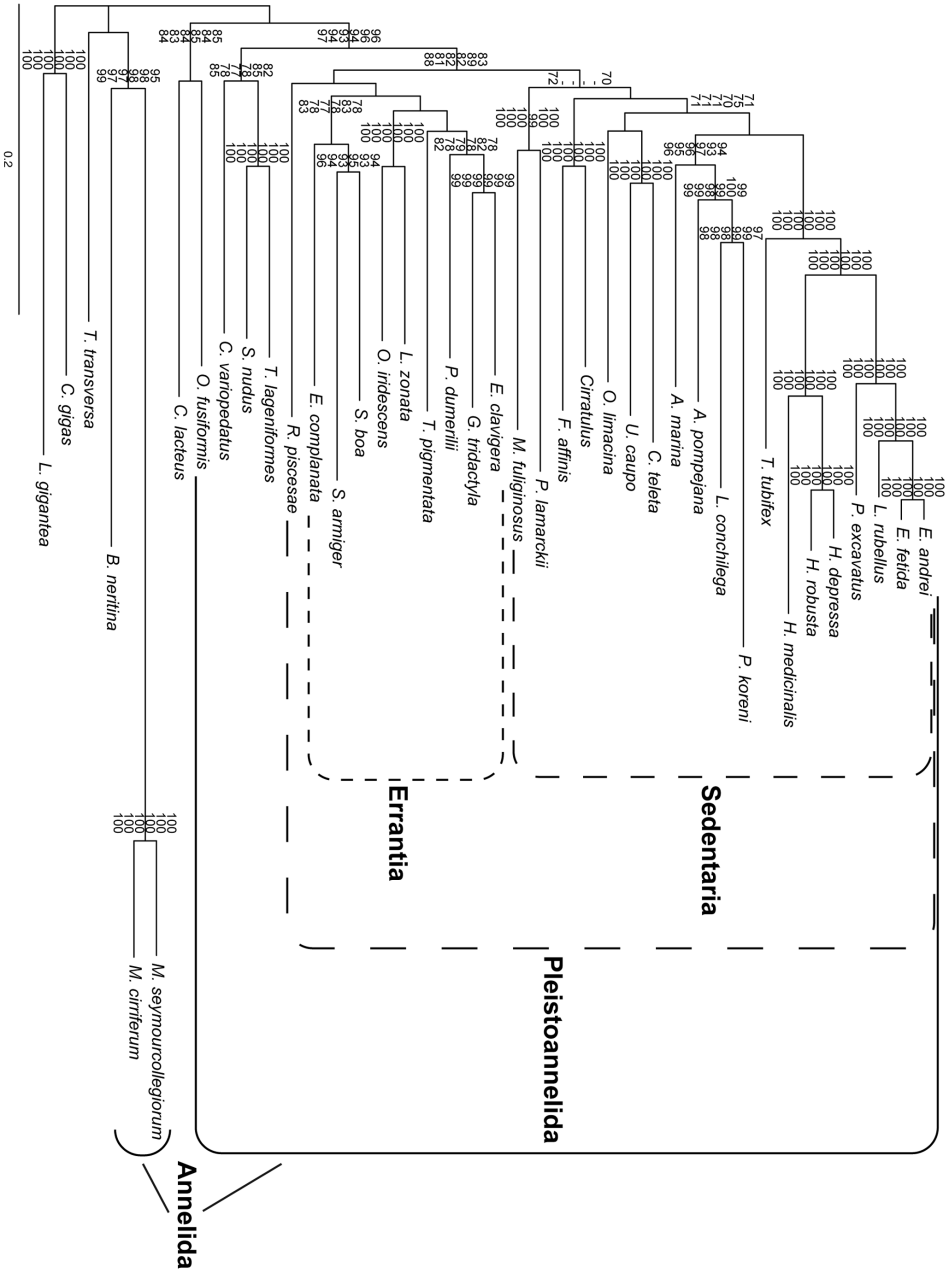
Phylogram of the ML analysis using the CPr dataset with contaminated sequences pruned. The dataset comprised 47,848 aa positions (-ln L = 667,263.00). Only bootstrap values ≥70 are shown at the branches. Below the bootstrap values of the analyses with the CPr dataset also the bootstrap values of the following analyses are shown in this order from top to down: contaminated sequences pruned and partition 24126 excluded (CPr24126, 47,658 aa positions, -ln L = 666,148.55), contaminated sequences and the paralogous ones of partition RPL24 pruned (CPrRPL24, 47,848 aa positions, -ln L = 666,917.26), contaminated sequences and the paralogous ones of partition 23636 pruned (CPr23636, 47,848 aa positions, -ln L = 667,034.41), contaminated sequences and the paralogous ones of partition 22680 pruned (CPr22680, 47,848 aa positions, -ln L = 666,522.29) and contaminated sequences pruned and partition 21904 excluded (CPr21904, 47,658 aa positions, -ln L = 664,271.95). Scattered lines indicate Errantia (short lines), Sedentaria (intermediate lines) and Pleistoannelida (long lines) and the solid lines Annelida. The scale bar indicates the number of substitutions per site.

Analyses of the concatenated dataset with the contaminated sequences pruned (dataset CPr) resulted in a tree topology similar to that for the AD dataset (Figs. 3 & 7). The only difference was the position of Opheliidae, but *L. conchilega* was still placed within Terebelliformia, *O. fusiformis* as sister to Nemertea, *G. tridactyla* sister to *Eulalia clavigera* and *T. pigmentata* as sister to three phyllodocidans. Thus, the artificial signal due to contamination was not able to alter the phylogenetic reconstruction by grouping *G. tridactyla* together with *T. pigmentata* and *L. conchilega* with *O. fusiformis*, respectively. However, the exclusion of the contaminated sequences had had an impact on the bootstrap support, it increased at several nodes. For example, the support for the nodes grouping *G. tridactyla* and *E. clavigera* together as well as *G. tridactyla*, *E. clavigera* and *P. dumerilii* increased from 70 to 99 and below 70 to 78, respectively. These are exactly the two nodes on the shortest path from *G. tridactyla* to *T. pigmentata* in the tree. Similarly, support increased for the nodes *L. conchilega*/*Pectinaria koreni* (88 to 97, Figs. 3 & 7), Terebelliformia (92 to 99), Arenicolidae/Terebelliformia (86 to 94), Pleistoannelida (78 to 83), Annelida (80 to 96), *O. fusiformis*/Nemertea (<70 to 85), which are all on the shortest path connecting *L. conchilega* and *O. fusiformis* in the tree. Hence, the contamination was able to reduce the nodal support at several nodes separating the affected taxa in the topology (Fig. 3). This is also reflected in leaf stability indices for *L. conchilega* and *O. fusiformis*, which clearly increased from the AD to the CPr dataset (Table 5). However, it is different for *T. pigmentata* and *G. tridactyla*, which did not change significantly. The change is similar to changes in other taxa not affected by the contamination, such as *Lumbricus rubellus*. The difference in the effect on the leaf stability indices is most likely due to the very different number of genes affected by contamination (nine versus one).

**Table 5:** Leaf stability indices of the taxa in the analyses of the AD (all data), CPr (contamination pruned) and CPPr (contamination and paralogy pruned) datasets.

Taxa	AD	CPr	CPPr
*Alvinella pompejana*	**0.936**	**0.948**	**0.941**
*Arenicola marina*	0.936	0.947	0.940
*Bugula neritina*	1.000	1.000	1.000
*Capitella teleta*	0.904	0.929	0.916
*Cerebratulus lacteus*	**0.864**	**0.863**	**0.915**
*Chaetopterus variopedatus*	0.950	0.963	0.931
*Cirratulus* sp	0.902	0.915	0.903
*Crassostrea gigas*	**0.897**	**0.877**	**0.916**
*Eisenia andrei*	0.945	0.953	0.946
*Eisenia fetida*	0.945	0.953	0.946
*Eulalia clavigera*	0.936	0.935	0.924
*Eurythoe complanata*	**0.879**	**0.883**	**0.766**
*Flabelligera affinis*	0.902	0.915	0.903
*Glycera tridactyla*	*0.936*	*0.935*	*0.924*
*Haementeria depressa*	0.945	0.953	0.946
*Helobdella robusta*	0.945	0.953	0.946
*Hirudo medicinalis*	**0.945**	**0.953**	**0.946**
*Lanice conchilega*	*0.870*	*0.948*	*0.941*
*Lottia gigantea*	**0.897**	**0.877**	**0.916**
*Lumbricus rubellus*	0.945	0.953	0.946
*Lumbrineris zonata*	0.934	0.930	0.922
*Malacoceros fuliginosus*	**0.882**	**0.900**	**0.888**
*Myzostoma cirriferum*	**0.986**	**0.977**	**0.995**
*Myzostoma seymourcollegiorum*	0.986	0.977	0.995
*Onuphis iridescens*	0.934	0.930	0.922
*Ophelia limacina*	0.886	0.896	0.888
*Owenia fusiformis*	***0.805***	***0.852***	***0.796***
*Pectinaria koreni*	0.936	0.948	0.941
*Perionyx excavatus*	0.945	0.953	0.946
*Platynereis dumerilii*	0.936	0.935	0.923
*Pomatoceros lamarckii*	**0.882**	**0.900**	**0.888**
*Ridgeia piscesae*	**0.762**	**0.765**	**0.787**
*Scoloplos armiger*	**0.930**	**0.931**	**0.810**
*Sipunculus nudus*	0.927	0.937	0.929
*Sthenelais boa*	**0.930**	**0.931**	**0.916**
*Terebratalia transversa*	0.869	0.859	0.922
*Themiste lageniformes*	0.927	0.937	0.929
*Tubifex tubifex*	0.945	0.953	0.946
*Typosyllis pigmentata*	*0.929*	*0.916*	*0.918*
*Urechis caupo*	**0.904**	**0.929**	**0.916**

Bold values indicate taxa affected by paralogy and italic by contamination.

Of the remaining 17 indicated clades 10 could be attributed to paralogy based on the blast results and seven did not show paralogy (Tables 1, 2,3, 4). For example, for partition 22431 the clade of *Arenicola marina*, *Capitella teleta* and *Urechis caupo* received a bootstrap value of 98 (Fig. 4B). However, for these three taxa as well as the two primer taxa *Lottia gigantia* and *Helobdella robusta* the blast returned NSA2 ribosome biogenesis homolog as the best hit (Table 1). Similarly, for 22539 the succinate dehydrogenase complex subunit D was found by the blast search for all investigated taxa (Table 1, Fig. 4D), for 22636 the eukaryotic translation initiation factor 5A2 (Table 2, Fig. 4F), for 22938 the NADH dehydrogenase (ubiquinone) 1α subcomplex subunit 13 (Table 2, Fig. 5C), for 23018 the proliferating cell nuclear antigen (Table 3, Fig. 5D) and for 23729 the trans-2,3-enoyl-CoA reductase (TECR) (Table 3, Fig. 5F). Hence, the strong support found for these clades could not be attributed to mistakenly assigned paralogs. No further analyses were conducted herein as the scope of this study was to investigate the impact of paralogous sequences. For the same reason, the sequences were left within the dataset because they were correctly placed within the ortholog group.

This left 10 clades in eight partitions, the strong support of which could be attributed to erroneous assignment of paralogs to an ortholog group. These cases could be assigned to two different classes. In the first class a primer taxon was among the taxa affected by paralogy and in the other class this was not the case. As described above, the first case was taken as evidence that for this partition the gene of the core ortholog set was already a mixture of paralogous sequences and not of orthologous ones and, hence, the complete partition should be excluded. This case was found in three partitions comprising five of the 10 clades. In partition 21904 two clades with strong support were detected: *U. caupo*/*L. gigantea* with a BP value of 99 and *Pomatoceros lamarkii*/*Myzostoma cirriferum*/*Crassostrea gigas* with a BP of 98 (Fig. 4A). For the sequences of the first clade the blast searches returned the Rho GDP dissociation inhibitor β as the best hit, whereas for the second clade it was the Rho GDP dissociation inhibitor α (Table 1). The results for the other two primer taxa were not so clear. Whereas *C. teleta* returned the Rho GDP dissociation inhibitor α, *H. robusta* returned Rho GDP dissociation inhibitor β. However, in contrast to *U. caupo* and *L. gigantea* the e value for the Rho GDP dissociation inhibitor α in the blast search of the *H. robusta* sequence was only slightly worse and the maximum score was even better for α than for β. Moreover, the sequences of *U. caupo* and *L. gigantea* showed clear signature amino acids, which separated them from the all other sequences in the alignment, but *H. robusta* did not. Nonetheless, at least one primer taxon was represented by a different paralog than the other primer taxa in this gene of the ortholog set. In 23816 a mixture of aldolase A and C results was returned as best hits in the three primer taxa and *Malacoceros fuliginosus*, but the alternative was always only slightly worse (Table 3, Fig. 6A). In contrast, in *R. piscesae* and *C. gigas* the alternative aldolase C was clearly worse than the best hit of aldolase A. However, given these uncertain results in the primer taxa this was also taken as evidence for an already affected gene in the core ortholog set. Similarly, in 24126 *L. gigantea* and *Themiste lageniformis* were affected, both returned different best hits from each other as well as to the two other primer taxa (Table 3, Fig. 6B).

In the other class primer taxa were not affected and always returned the same best hits in the blast searches. This class comprised five clades in five partitions. In 22433 *Scoloplos armiger*, *Sthenelais boa* and *Eurythoe complanata* grouped together with a BP value of 100 (Fig. 4C). The blast searches returned for these three sequences the proteasome subunit α2 (PSMA2), whereas for the primer taxa the proteasome subunit α8 (PSMA8) was returned (Table 1). Hence, in this case the PSMA2 sequences of these three taxa were erroneously assigned to the ortholog group of PSMA8. Therefore, in this case only these three sequences were pruned and not the entire partition. The results were similar in the other four cases and were treated similarly. In 22606 *O. fusiformis* and *C. lacteus* grouped together with a BP value of 100 (Fig. 4E). The blast searches found for these two sequences centrin, EF-hand protein 3 (CETN3) as the best hit and centrin, EF-hand protein 2 (CETN2) for the primer taxa (Table 2). In 22680 *P. lamarckii* and *Alvinella pompejana* grouped together with a BP value of 99 (Fig. 5A). The blast searches found for these two sequences heat shock 70 kDa protein 1A as the best hit and heat shock 70 kDa protein 8 for the primer taxa (Table 2). In 23636 *Hirudo medicinalis* and *A. pompejana* grouped together with a BP value of 99 (Fig. 5E). The blast searches found for these two sequences NADH dehydrogenase (ubiquinone) 1α subcomplex, assembly factor 2 as the best hit and NADH dehydrogenase (ubiquinone) 1α subcomplex 12 for the primer taxa (Table 3). In RPL24 *L. conchilega* and *Eisenia fetida* grouped together with a BP value of 96 (Fig. 6C). The blast searches found for these two sequences RPL24 domain containing 1 as the best hit and RPL24 for the primer taxa (Table 4).

**Figure 8:**
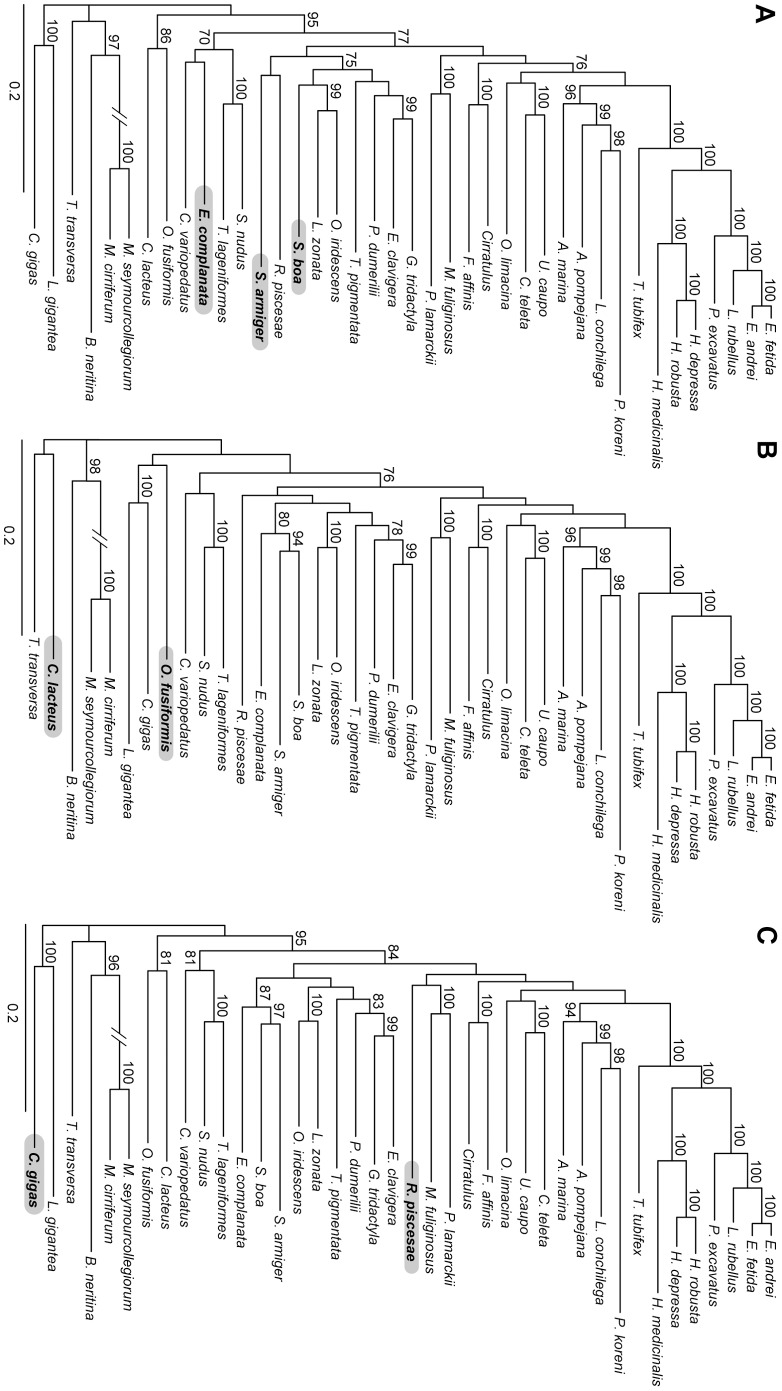
Phylograms of ML analyses using datasets with contaminated and some paralogous sequences pruned. (A) contaminated sequences and the paralogous ones of partition 22433 pruned (CPr22433, 47,848 aa positions, -ln L = 666,010.86). (B) contaminated sequences and the paralogous ones of partition 22606 pruned (CPr22606, 47,848 aa positions, -ln L = 666,512.82). (C) contaminated sequences pruned and partition 23816 excluded (CPr23816, 47,490 aa positions, -ln L = 663,269.44). Scattered lines indicate Errantia (short lines), Sedentaria (intermediate lines) and Pleistoannelida (long lines) and the solid lines Annelida. The scale bar indicates the number of substitutions per site. Taxa placed differently in Fig. 7 are in bold and highlighted with a grey box.

To assess the impact of the paralogous sequences in the analyses of the concatenated dataset, the affected sequences or partitions were pruned in turn from the CPr dataset. In two of the analyses the pruning had had a strong impact on the results and affected groups strongly supported in the CPr and/or AD dataset. Pruning the sequences of *S. armiger*, *S. boa* and *E. complanata* in partition 22433 had had an impact on the position of these three taxa. In the analyses of the CPr dataset these three taxa were grouped together with a BP value of 78 and within this clade *S. armiger* and *S. boa* grouped together with a BP value of 94 (Fig. 7). However, in the analyses of the CPr22433 dataset *S. boa* was placed within a clade which also comprised the other phyllodocidan taxa and Eunicida with a BP value of 75 (Fig. 8A). *S. armiger* was together with *R. pisceseae* as sister to this clade, but with low nodal support values. *E. complanata* was placed outside Pleistoannelida within a clade comprising also Chaetopteridae and Sipuncula with a BP value of 70. Thus, the strong support for especially the sistergroup relationship of *S. armiger* and *S. boa* based on the AD and CPr dataset (Figs. 3 & 7) stemmed from the paralogous sequences for these two taxa. Similarly, the analyses based on the dataset in which the sequences of *O. fusiformis* and *C. lacteus* of the partition 22606 were pruned (CPr22606) did not recover the sistergroup relationship of these taxa (Fig. 8B) as found in the AD and CPr data with BP values below 70 or of 85, respectively (Figs. 3 & 7). Instead *C. lacteus* was sister to *T. transversa* and *O. fusiformis* to Mollusca, but both with only low nodal support values (Fig. 8B). Thus, as in the previous case support for the sistergroup relationship of *C. lacteus* and *O. fusiformis* stemmed from paralogous sequences.

In the other six cases there was no impact at all or only a single taxon was placed differently, but the placement of this taxon had not been strongly supported in the first place in the analyses based on the AD or CPr datasets. For example, excluding partition 23816 from the CPr dataset (CPr23816) placed *R. piscesae* within Sedentaria with low nodal support (Fig. 8C). This placement was similar to the analyses of Struck et al. [26], which also placed the siboglinid *R. piscesae* in Sedentaria. The placement of *R. piscesae* in the AD and CPr datasets as sister to Errantia was also only supported by low BP values. In contrast, *C. gigas*, which was also affected by paralogy in this partition, was not placed differently and the BP value was still 100 for the sistergroup relationship to the other mollusk *L. gigantea* in the analyses (Figs. 3, 7 & 8C). Similarly, either pruning of affected sequences in 23636 or 22680 or excluding the partitions 24126 or 21904 resulted in a different placement of *T. transversa*. Instead of being a weakly supported sistergroup to Myzostomidae/*B. neritina* (Figs. 3 & 7) *T. transversa* was the weakly supported sister of *C. lacteus*/*O. fusiformis* (BS <70, not shown). Interestingly, *T. terebratalia* was present neither in the excluded partitions 24126 and 21904 nor in the partitions 23636 and 22680 from which sequences were pruned (Figs. 4A, 5A, 5E & 6B). Thus, the different placement of *T. terebratalia* could not be related to paralogy, but to generally low phylogenetic signal in the dataset for a robust placement of *T. terebratalia*. Moreover, in all six cases BP values of strongly supported nodes were not substantially altered by the pruning procedures (Figs. 7 & 8C).

**Figure 9:**
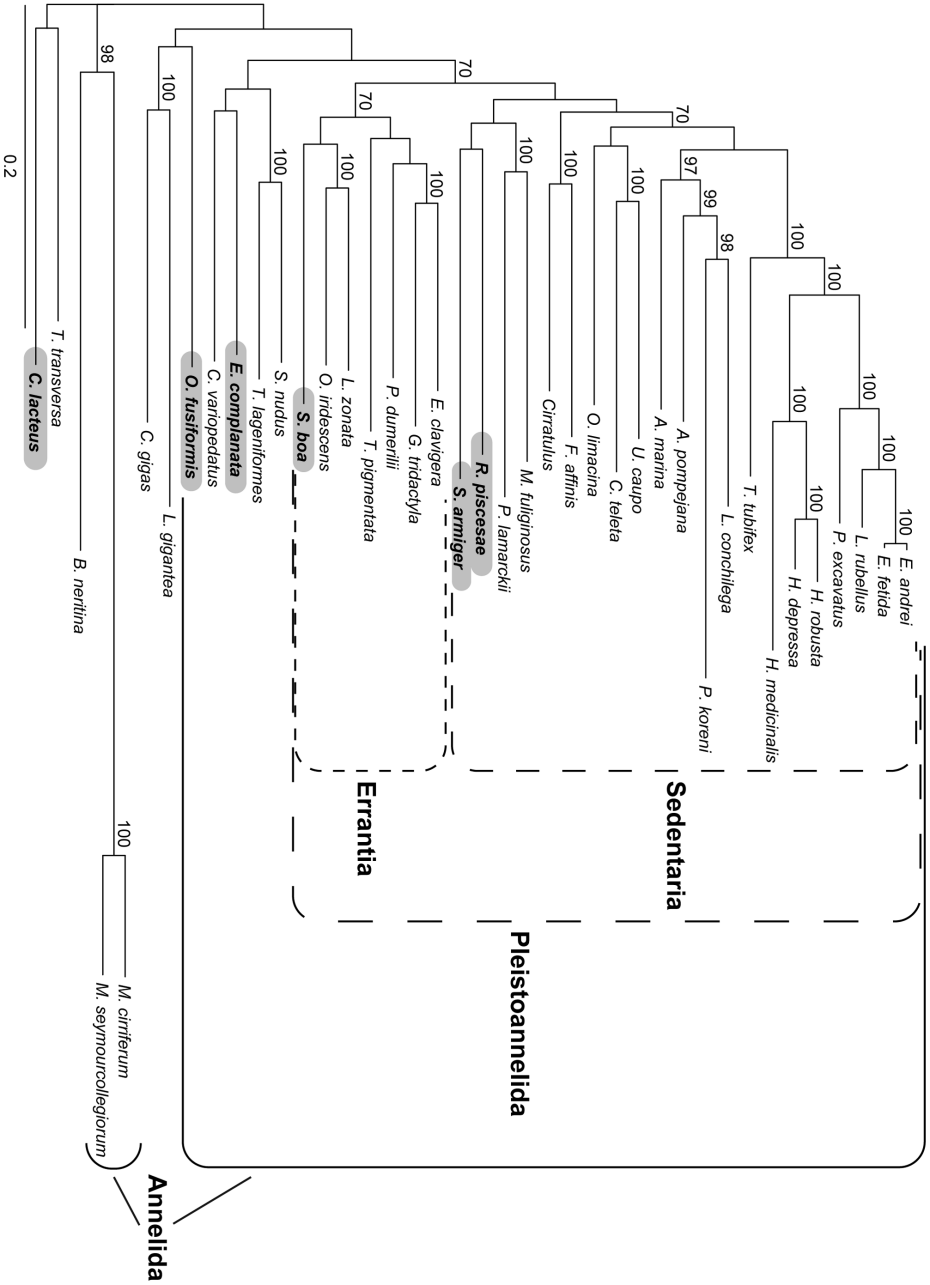
Phylogram of the ML analysis using the dataset with all contaminated and paralogous sequences pruned. (CPPr, 47,225 aa positions, -ln L = 655,792.81). Scattered lines indicate Errantia (short lines), Sedentaria (intermediate lines) and Pleistoannelida (long lines) and the solid lines Annelida. The scale bar indicates the number of substitutions per site. Taxa placed differently in Fig. 7 are in bold and highlighted with a grey box.

In contrast to the AD and CPR results, the analyses of the dataset in which all affected sequences or, if appropriate, partitions were excluded (CPPr) showed *S. armiger* to be sister to *R. piscesae* and both were placed within Sedentaria as sister to *M. fuliginosus*/*P. lamarckii*, but with low nodal support values only (Fig. 9). *S. boa* was still part of Errantia, though BP support for Errantia was only 70 (Fig. 9). *E. complanata* was not placed within Errantia or Pleistoannelida, but as sister to Chaetopteridae within a clade also comprising Sipuncula. However, nodal support was low again. *O. fusiformis* was now sister to Mollusca and *C. lacteus* to *T. transversa*, but with low nodal support for both groupings.

Again this is in part reflected in the changes of the leaf stability indices from the CPr to CPPr dataset (Table 5). Except for *S. boa* and *R. piscesae*, all taxa on which paralogy had an impact in the phylogenomic analyses exhibited a difference of above 0.05. For *O. fusiformis* the value strongly decreased, whereas it increased for *C. lacteus*, indicating that *O. fusiformis* was drawn towards *C. lacteus* by the paralogy and not vice versa. Similarly, the value for *S. boa* stayed constant, whereas it strongly decreased for *E. complanata* and *S. armiger*. Again this indicates that *E. complanata* and *S. armiger* were drawn towards *S. boa* and not vice versa. The change of the value for *R. piscesea* is in the range of below 0.04 for the taxa which were either not affected by paralogy at all or whose phylogenetic position was not affected by paralogy. Of these taxa only *T. transversa* increased by a value above 0.05. Hence, *T. transversa* was the only taxon, which was indirectly affected by the paralogy.

## Discussion

The analyses herein showed that paralogous sequences had had an impact on the support for the position of some taxa (i.e., *Scoloplos armiger* (Orbiniidae), *Sthenelais boa* (Aphroditiformia), *Eurythoe complanata* (Amphinomidae), *Owenia fusiformis* (Oweniidae), and *Cerebratulus lacteus* (Nemertea)) in the concatenated dataset, whereas for other taxa (i.e., *Urechis caupo* (Echiura), *Pomatoceros lamarckii* (Sabellida), *Malacoceros fuliginosus* (Spionidae), *Hirudo medicinalis* (Clitellata), *Alvinella pompejana* (Terebelliformia), *Myzostoma cirriferum* (Myzostomidae), *Crassostrea gigas* (Mollusca), *Lottia gigantea* (Mollusca)) it had none. *Ridgeia piscesae* (Siboglinidae) is intermediate between these two cases as it is placed differently, but its placement is not strongly supported regardless of the inclusion or exclusion of the *R. piscesae* sequence affected by paralogy in partition 23816.

The difference between these two sets of taxa (impact vs. no impact) is that the taxa on which the paralogous sequences had had no impact in the concatenated dataset were not the only representatives of a higher taxon, for which already morphological data or molecular studies based on a single or few genes provided stronger support. For example, the sistergroup relationship of Echiura, represented by *U. caupo* herein, and Capitellidae, represented by *Capitella teleta* herein, was strongly supported already in previous molecular studies (e.g., [2], [49], [51]). Similarly the close relationship of Sabellida, represented by *P. lamarckii* herein, and Spionidae, represented by *M. fuliginosus* herein, was consistently recovered in previous molecular studies (e.g., [2], [49]). Monophyly of Clitellata, of Myzostomidae, of Terebelliformia as well as of Mollusca is well supported by both morphological and molecular data (e.g., [48], [60], [68], [74], [75], [76], [77]). All four taxa are represented by more than one taxon in these analyses. Hence, the phylogenetic signal for these monophyletic groups throughout the entire dataset overwhelmed the artificial signal due to the paralogous sequences. For example, placement of *H. medicinalis* within Hirudinea (Clitellata) is strongly supported by 57 partitions (i.e. BP value above 94) or of *M. cirriferum* within Myzostomidae by 56 partitions, in contrast to the single partitions which showed a paralogous sequence for these two taxa (i.e., *H. medicinalis* sequence of partition 23636 and *M. cirriferum* sequence of 21904). Moreover, not only the placement of the affected taxon within the specific group (e.g., *H. medicinalis* in Clitellata), but also the placement of the entire specific group was not different if the affected sequences were excluded. On the other hand, if there was an impact due to paralogy it was always for taxa which were the only representatives of a higher taxon, for which previous studies had gathered stronger support (i.e., *S. armiger* for Orbiniidae, *S. boa* for Aphroditiformia, *E. complanata* for Amphinomidae, *O. fusiformis* for Oweniidae, *R. piscesae* for Siboglinidae and *C. lacteus* for Nemertea) (e.g., [50], [78], [79], [80], [81]). Thus, if a taxon is the only representative of a previously strongly supported higher taxon such as a polychaete family, paralogous sequences can have a strong impact on its position in the reconstructed tree even in phylogenomic datasets, whereas a better taxon representation of such a taxon is able to ameliorate the negative impact of a paralogous sequence on the reconstruction of the complete dataset.

This conclusion is further substantiated by the fact that the effect of the paralogous sequences of *O. fusiformis* and *C. lacteus* in partition 22606 was ameliorated by substantially increasing the number of outgroup taxa to 17, which also included two additional nemerteans, without pruning of affected sequences (preliminary analyses not shown). In these analyses *O. fusiformis* was within Annelida as sister to Chaetopteridae with a BP value of 98, whereas *C. lacteus* was placed within a monophyletic Nemertea supported by a BP value of 100.

The impact of paralogy on other phylogenomic studies has still to be assessed thoroughly, but other misleading factors such as long branch attraction or compositional biases might be actually more influential in these studies, as the relevant taxonomic groups are usually represented by more than one taxon (e.g., [23], [25], [27], [28], [82], [83]). In phylogenomic studies of bilaterian taxa, for example, only taxa such as Gastrotricha, Gnathostomulida, Phoronida, Brachiopoda, Priapulida, Kinorhyncha and Nematomorpha were represented by merely a single taxon with often a small EST library, but more strongly supported surprising clades among the bilaterian phyla like the clade of *O. fusiformis* and *C. lacteus* described here could not be observed [23], [25]. In comparison to Dunn et al. [23] Hejnol et al. [25] increased the taxon sampling of long-branched Acoelamorpha taxa, with the result that these were placed as sister to Bilateria instead of within long-branched Platyzoa. This also affected the position of long-branched Myzostomidae, which were placed within Annelida instead of Platyzoa. Moreover, a following study specifically addressing long-branch issues found a placement of Acoelomorpha within Deuterostomia [43]. Thus, the position of Acoelomorpha is more likely to be influenced by increased substitution rates than paralogy. In a recent phylogenomic study of ecdyszoan relationships also addressing the aspect of increased substitution rates at least two taxa represent most relevant taxa (i.e., Hexapoda, Crustacea, Myriapoda, Chelicerata, Onychophora, Tardigarda, and Nematoda), but Nematomorpha, Kinoryncha and Priapulida were represented by a single taxon each [82]. Moreover, some of the EST libraries in that study were as small as in the analysis herein. Nonetheless, overall it can be expected that support for a clade based only on paralogous sequences in the grouped taxa is not very likely in most recent phylogenomic studies. However, as phylogenomic studies become more common nowadays and are used for very different phylogenetic questions, the analyses herein show that the issue of paralogy should not be neglected in the design of the study.

Although an increased taxon sampling most likely ameliorates the impact of paralogous sequences on phylogenomic studies, the artificial signal would still be present in the dataset. Moreover, for some taxa it might not be easily achievable to obtain another closely related sister-taxon fitting the criterion that that relationship already gathered some support by morphological data and/or molecular studies based on a single or few genes. A more conservative and rigid procedure would be to detect such paralogous sequences and prune them entirely from the dataset as was done herein. However, as phylogenomic studies are easily based on hundreds or thousands of genes an automatic detection and pruning would be time-saving and it would be unlikely that a case is overseen if hundreds or thousands of partition trees are checked by eye only. Thus, are there certain criteria or procedures, which would allow the secure detection of paralogs?

Herein a screening procedure based on bootstrap support was chosen to detect suspicious cases, as at the taxonomical level of this study it was known that in single gene analyses nodal support values for deep-level relationships are usually low. High bootstrap support, thus, could be an indicator of potential paralogy. However, even filtering clades such as Clitellata, for which strong support can be assumed in single gene analyses (e.g., [5], [84], [85], [86]), in seven of the 17 indicated clades in 14 partitions no wrongly assigned paralogous sequences were found (Tables 1, 2,3, 6). Hence, the strong observed support in these cases could be due to either a true phylogenetic signal or to other biases such as compositional heterogeneity, increased substitution rates or shared missing data misleading the phylogenetic reconstruction of the single partition. With respect to it being a true phylogenetic signal it should be noted, however, that none of these clades were found in the analyses of the different concatenated datasets except for the clade of *Terebratalia transversa* and *C. lacteus*, which can be found in partition 23018 (Table 3, Fig. 5D) and in some of the trees based on pruned datasets as well (Figs. 8B and 9). With respect to an automatic screening and pruning procedure of wrongly assigned paralogous sequences from the dataset, only using the bootstrap value would not work, as there is substantial overlap between cases not due to paralogy and the ones due to paralogy (Table 6). Nonetheless, of the detected clades consisting of paralogous sequences most had a bootstrap support of 99 or 100.

**Table 6:** Comparison of bootstrap values and branch length measurements for the detected clades by the screening, which are affected by paralogy or not.

partition		bootstrap	branch	branch
		value	length^1^	ratio^2^
**no paralogs**				
22431	A	98	0.0379	0.98
22539	A+B	96	0.1997	1.22
23729	A	96	0.1466	1.51
22938	A	98	0.3748	2.37
22636	A	98	0.2843	2.46
23018	A	98	0.1003	2.57
22539	A	100	0.6049	3.70
**potential paralogs**				
23816	A	96	0.0834	0.77
24126	A	97	0.1497	1.57
21904	B	98	0.2313	1.87
21904	A	99	0.2362	1.91
22606	A	100	0.9936	4.40
23816	B	100	0.543	5.00
22680	A	99	0.2545	5.24
RPL24	A	96	1.7501	7.45
23636	A	99	4.1402	10.17
22433	A	100	4.1762	13.40

1 length of the internal branch leading to the detected clade.

2 ratio of the length of the internal branch leading to the detected clade to the average of all internal branch lengths.

Instead of or in combination with nodal support values the length of the branch leading to the group of paralogous sequences could also be considered as an indicator of paralogous sequences. If the gene duplication event between the two paralogs occurred very early in the evolution of these taxa (e.g., predating the origin of Annelida) both paralogs could have accumulated enough genetic differences from one another in comparison to the intra-paralog genetic distances within each paralog. However, comparing the two cases (i.e., affected by paralogy or not) it can be observed that both the actual length of the branch leading to the clade in question and the ratio of this length to the mean of all other internal branch lengths is overlapping (Table 6). The values of the actual branch length range from 0.0389 to 0.6049 for the clades for which no paralogy could be detected, and the values of the ratio from 0.98 to 3.70. Similarly, for the clades with paralogy issues the corresponding values range from 0.0834 to 4.1762 and from 0.77 to 13.40. Hence, an automatic screening and pruning procedure of wrongly assigned paralogous sequences from the dataset using only or in addition branch length or genetic distance estimations would not work either.

A suitable approach would be to conduct blast searches of suspicious sequences, for example those detected by a nodal support or branch length screen, as well as of sequences of primer taxa against the transcriptome of a reference taxon, which is not part of the primer taxon set, as it has been done herein. Using local databases of such taxa and blast searches, such analyses could also be automated. However, more than one reference taxon should be used in turn as was done herein. Otherwise a case of paralogy might be missed. For example, the blast searches of the sequences of *O. fusiformus*, *C. lacteus, L. gigantea*, *H. robusta* and *C. teleta* of partition 22606 returned in the blast searches against *B. taurus* the same hits (CETN1, NM_001079596) and only the blast search against *B. floridae* was able to reveal the paralogy issue.

**Figure 10:**
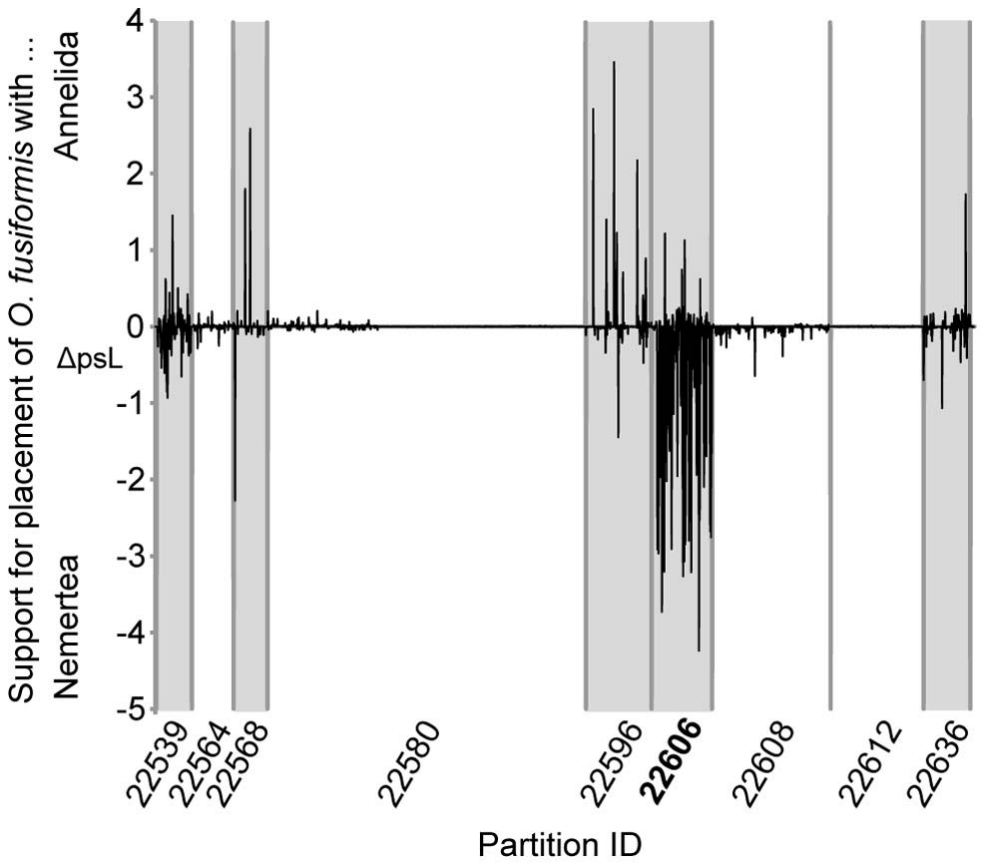
Plot of per-site likelihood differences (ΔpsL) for the alignment region around the partition 22606. Partition ID’s are indicated, and grey highlights partitions in which sequence information for *Owenia fusiformis* was present. Partition 22606 is in bold.

Instead of checking for paralogy across all taxa before the phylogenetic analyses of the concatenated data, another strategy after the analyses could be to test if the support for a specific clade of interest stems only from paralogy. For example, the surprising clade of *O. fusiformus* and *C. lacteus* could have been tested in this way. If only the clades with strong nodal support, which contained also *O. fusiformis*, had been selected both the nine clades indicating the contamination problem in the EST library of *L. conchilega* and the clades in partitions 22606 and 22539 would have been indicated (Tables 1–4). Of these only the partition 22606 would have had also *C. lacteus* in the clade. Vice versa, screening for *C. lacteus* would have revealed the partitions 22606 and 23018 as possessing clades with strong nodal support containing *C. lacteus* and only partition 22606 would have additionally contained *O. fusiformis*. This would indicate that support of the clade of *O. fusiformus* and *C. lacteus* might mainly stem from this partition and, hence, this partition should be further explored with respect to artificial signal such as paralogous sequences. If single-partition analyses are to be avoided in such a case another strategy is possible if two alterative hypotheses can be compared against each other. The difference between the per-site likelihoods of the two hypotheses (ΔpsL) can be calculated and plotted for each site. In that way it can be assessed if the support for the hypotheses is evenly distributed along the alignment or concentrated in certain genes. Taking the *O. fusiformis*/*C. lacteus* example again, such a plot shows that the support for this clade is strongly concentrated in the partition 22606 of the alignment (Fig. 10). Smith et al. [28] used such an approach comparing the alternative hypotheses of Monoplacophora with Polyplacophora or Cephalopoda. They could show that the support for the clade of Monoplacophora and Cepholapoda was evenly distributed across the alignment and not concentrated in a certain gene as shown in Fig. 10. Evans et al. [42] also invoked a per-site likelihood approach to explore the signal for the two alternative hypotheses of Myxozoa placed within Cnidaria or as sister to Bilateria. The plot also showed that the support for both hypotheses was evenly distributed along the alignment and not concentrated in specific genes. Hence, an influence of paralogous sequences on these hypotheses could be excluded.

The degree of erroneously assigned paralogous sequences in the analysis can also be reduced already in the orthology prediction. The scenario discussed in the Introduction, that the reciprocal lack of the two paralogs of a gene family in corresponding taxa sets can lead to the wrong assignment of paralogous sequences to an ortholog core gene (Fig. 1), has been found to be true in the analyses herein. For example, the ortholog gene of partition 22433 belongs to the gene family of the proteasome subunit α family and more specifically it is the member PSMA8. This gene is present in all three primer taxa of these analyses (Table 1). However, it was lacking in the EST libraries of *S. armiger*, *S. boa* and *E. complanata*. In the HaMStR hidden Markov search the member PSMA2 of the gene family was found in these three taxa instead. The transcriptome of the primer taxon *H. robusta* was used as the reference taxon for the back-blast of these sequences and returned as a best hit PSMA8 and not PSMA2. Thus, reciprocity was fulfilled and the PSMA2 sequences of *S. armiger*, *S. boa* and *E. complanata* were “correctly” assigned to the PSMA8 group given the algorithm. The transcriptome of *H. robusta* just lacked PSMA2. This was proven by a blast of the *B. taurus* PSMA2 sequence, which was returned as the best hit in the blast searches against *B. taurus* for the sequences of *S. armiger*, *S. boa* and *E. complanata* (Table 1), against the transcriptome of *H. robusta* and which also returned *H. robusta* PSMA8 as the best hit with an e-value of e^−41^.

There are two strategies to ameliorate the effect of reciprocal lack by filling one of the empty gaps in Fig. 1. If just one of the lacking sequences could be provided, the wrong assignment would not happen as either the correct orthologous sequence is found or reciprocity is not fulfilled. The first strategy is to sequence more reads of the non-primer taxon to generate a better-covered transcriptomic library and, thus, increase the likelihood that the gene searched for is actually represented in the library. For example, the EST libraries of *O. fusiformis*, *L. conchilega*, *E. fetida*, *S. armiger*, *S. boa* and *E. complanata* were small with no more than 2,000 reads. On the other hand, the libraries of *C. lacteus* with 5,127, *P. lamarckii* with 4,132, *A. pompejana* with 142,322, and *H. medicinalis* with 26,833 reads were not small. Thus, to increase the quality of the EST library with respect to coverage of represented genes not only more reads are necessary, but they should also come from different developmental stages and tissues. However, this is not always possible for non-model organisms, which are not kept in the laboratory, but are taken out of their natural habitats. Nonetheless, with the new sequencing technologies much deeper sequenced transcriptomes for non-model organisms are becoming more common nowadays and, thus, the wrong assignment of paralogous sequences will become less likely. On the other hand, the possibility still exists that the gene in question has been actually lost in the corresponding taxon and its absence is not just due to lack of enough sequence data.

The other option is to fill the gap at the side of the primer taxa so that the reciprocity criterion is not fulfilled and, hence, the paralogous sequence is not assigned to the wrong group. This can be most easily achieved by using more than one primer taxon as reference taxa. For example, instead of using only *H. robusta* as in the analyses presented herein, the other primer taxa *C. teleta*, *L. gigantea*, *Schistosoma mansoni*, *Daphnia pulex*, *Apis mellifera* and/or *Caenorhabditis elegans* could be used as well (e.g., using the strict option in HaMStR). The probability that all of these seven primer taxa lack the same paralogous sequence is extremely small. Thus, in at least one of the reference taxa the paralogous sequence would be the better hit than the gene sequence actually searched for. For example, in blast searches of the *S. armiger*, *S. boa* or *E. complanata* sequence of partition 22433 against the transcriptome of *C. teleta* the *C. teleta* PSMA2 sequence was returned as the best hit with an e-value of e^−117^, e^−80^ or e^−43^ and the PSMA8 sequence only as second or third best hit with an e-value of e^−41^, e^−29^ or e^−9^, respectively. Hence, reciprocity would not be fulfilled and the paralogous sequence would not be assigned to this group as it had happened using only *H. robusta*.

Finally, in the orthology prediction using HaMStR the representative option was chosen herein as this was the option used by Struck et al. [26]. Using this option up to three best hits of the searches based on hidden Markov models are kept for the blast search against the reference taxon/taxa. If two or more of these hits fulfill the reciprocity criterion and match non-overlapping parts of the reference protein, the sequences of these hits will be concatenated into a single sequence. This has the advantage that more sequence information is kept during the orthology prediction, but it also risks that chimera of paralogous and orthologous sequences are generated. The ability to detect such chimera using approaches based on the entire gene, like the one used herein, is reduced as mixed signals are present in the gene. Approaches, which undo such concatenations in the course of the analyses, would be better suited to detect such cases of paralogy.

### Conclusions

Wrongly assigned paralogous sequences can have an impact on the phylogenetic reconstruction even in phylogenomic datasets, but only when the affected taxa were the only representatives of a generally and previously supported higher taxon such as a polychaete family. Hence, the impact of paralogous sequences on other recent phylogenomic studies might be neglectable, but this has not been explicitly assessed except for two specific instances regarding the position of Monoplacophora and Myxozoa, respectively. *A priori* detection of wrongly assigned paralogous sequences should combine a screening of single-partition analyses based on either nodal support values, if it can be assumed that high bootstrap values in single genes are not very likely, or on the length of the branch leading to the affected taxa and blast searches against transcriptomic databases of more than one reference taxon. *A posteriori* approaches can be used if only specific clades, which were retrieved in the analyses of the data, are to be tested either by using an approach similar to the *a priori* approach or by comparing two alternative hypotheses using differences in per-site likelihoods. Finally, increasing the sizes of the EST libraries in the analyses will also decrease the likelihood to wrongly assign paralogous sequences to a group of orthologous sequences from the same gene family. Moreover, in top-down orthology determination processes such as HaMStR using more than one reference taxon will also decrease this likelihood.
